# Tau interactome analyses in CRISPR-Cas9 engineered neuronal cells reveal ATPase-dependent binding of wild-type but not P301L Tau to non-muscle myosins

**DOI:** 10.1038/s41598-019-52543-5

**Published:** 2019-11-07

**Authors:** Xinzhu Wang, Declan Williams, Iris Müller, Mackenzie Lemieux, Ramona Dukart, Isabella B. L. Maia, Hansen Wang, Amanda L. Woerman, Gerold Schmitt-Ulms

**Affiliations:** 1 Tanz Centre for Research in Neurodegenerative Diseases, University of Toronto, Ontario, M5T 2S8 Canada; 2 Department of Laboratory Medicine & Pathobiology, University of Toronto, Ontario, M5S 1A8 Canada; 3 Department of Neurology, University of California San Francisco, California, 94158 USA

**Keywords:** Protein-protein interaction networks, Neurodegeneration, Molecular neuroscience

## Abstract

Protein interactions of Tau are of interest in efforts to decipher pathogenesis in Alzheimer’s disease, a subset of frontotemporal dementias, and other tauopathies. We CRISPR-Cas9 edited two human cell lines to generate broadly adaptable models for neurodegeneration research. We applied the system to inducibly express balanced levels of 3-repeat and 4-repeat wild-type or P301L mutant Tau. Following 12-h induction, quantitative mass spectrometry revealed the Parkinson’s disease-causing protein DJ-1 and non-muscle myosins as Tau interactors whose binding to Tau was profoundly influenced by the presence or absence of the P301L mutation. The presence of wild-type Tau stabilized non-muscle myosins at higher steady-state levels. Strikingly, in human differentiated co-cultures of neuronal and glial cells, the preferential interaction of non-muscle myosins to wild-type Tau depended on myosin ATPase activity. Consistently, transgenic P301L Tau mice exhibited reduced phosphorylation of regulatory myosin light chains known to activate this ATPase. The direct link of Tau to non-muscle myosins corroborates independently proposed roles of Tau in maintaining dendritic spines and mitochondrial fission biology, two subcellular niches affected early in tauopathies.

## Introduction

More than 30 years ago the Tau protein was first identified in cellular deposits that accumulate in Alzheimer disease (AD)^1^. Since then, Tau has been implicated in numerous neurodegenerative diseases, collectively known as “tauopathies”, that include AD, progressive supranuclear palsy (PSP), corticobasal degeneration (CBD), and frontotemporal dementia with parkinsonism-17 (FTDP-17)^2^. Tau is understood to be critical for cellular toxicity to manifest in these diseases^3–9^. Moreover, mutations in the microtubule-associated protein Tau (*MAPT*) gene are sufficient to cause FTDP-17 with a middle-age onset independently of amyloid β plaques or other known protein aggregates^2,10–12^. The objective to delineate Tau interactions has therefore become a focus of mechanism-based disease intervention efforts. Yet, to date, the specific molecular underpinnings that mediate Tau-dependent perturbations to the cell are not understood. We recently reported on a systematic analysis of Tau protein interactions in a neuroblastoma cell line, which revealed that Tau binds predominantly to the ribonucleoproteome, chaperones, the proteasome, histone complexes, and members of the 14-3-3 protein family^13^. When compared with four-repeat wild-type (4R^wt^) Tau expressing cells, the expression of P301L mutant (4R^P301L^) Tau exhibited reduced binding to a subset of heat-shock proteins and the proteasome in this prior study. However, a confounder of this work was that it relied on the stable expression of plasmid-encoded 4R Tau in dividing cells, providing little control over isoform expression levels and restricting the analysis to studying steady-state interactions of the Tau bait protein.

Neuronal brain Tau exists in six isoforms caused by the presence or absence of one or two 29 amino acid insertions in the N-terminal half of the protein and the alternative inclusion of a 31 amino acid repeat sequence in the domain responsible for Tau’s binding to microtubules^14^. The inclusion or omission of the latter repeat domain determines whether Tau has three or four of these domains, with P301L residing in the fourth repeat. Whereas N-terminal insertions do not seem to impact disease propensity, a relatively balanced ratio of 3R versus 4R Tau is observed in healthy neuronal cells^2,15^. Human neuroblastoma cell models in use for studying Tau biology (SH-SY5Y or IMR-32) are known to express only low levels of endogenous Tau. Although overexpression of 4R Tau has repeatedly been observed to be mildly toxic^15^, a majority of studies preceding this work were based on 4R overexpressing cells.

Recent advances in genome-editing technology have reduced the time and costs needed to generate gene-engineered cell models. Despite these advances, it typically still requires considerable resources to build experimental models for studying a protein-of-interest in a relevant paradigm. While an increasing number of inducible clones have become available, when we embarked on this project approximately two years ago, to the best of our knowledge, no such tools were available for the study of brain proteins in a flexible and site-specific manner that is minimally confounded by the effects of random transgene integration. Hence, it would be desirable to be able to rapidly generate a human diploid cell model that can be fully differentiated into neurons, astrocytes or oligodendrocytes and can be induced to (1) express a protein-of-interest, (2) visualize its subcellular distribution, and (3) study its interactions and post-translational modifications.

To address these shortcomings, we used CRISPR-Cas9 gene editing technology to insert foundation cassettes harboring an antibiotic resistance gene flanked by lox sites, which are later used as acceptor sites to efficiently integrate large inducible constructs, into the human adeno-associated virus integration site 1 (*AAVS1*) genomic safe harbor of two cell lines with useful characteristics for Alzheimer disease and Tau-related research, namely IMR-32 neuroblastoma cells^16–18^ and a neuroprogenitor cell line derived from the ventral mesencephalon (ReN VM)^19^, hereafter referred to as IMR and ReN cells. With these acceptor cell clones in hand, we could direct inducible expression cassettes into the primed *AAVS1* loci using Cre recombinase, bypassing the size limit CRISPR-Cas9 gene editing has for integrating large transgenes. We initially tested the system in dividing neuroblastoma IMR cells with constructs encoding the inducible expression of 3R and 4R wild-type (or P301L mutant) Tau fused at the C-terminus to an enhanced green fluorescent protein (EGFP). We hypothesized that the inducible wild-type (3R^wt^/4R^wt^) and P301L mutant (3R^wt^/4R^P301L^) Tau might affect cellular outcomes by interacting with different protein partners. Hence, we compared the interactomes of 3R^wt^/4R^wt^ and 3R^wt^/4R^P301L^ using quantitative mass spectrometry. This first set of data confirmed previously reported Tau binders but also produced several new candidate Tau interactors, including DJ-1, a protein genetically linked to Parkinson’s disease. We next moved away from mitotically active cells to study Tau interactions in ReN cells, which we differentiated into co-cultures of non-dividing neurons and glia. Stringent interactome analyses undertaken with this paradigm put a spotlight on a novel interaction between wild-type Tau and non-muscle myosins that relied on ATPase activity of these cytoskeletal motors and was diminished in cells expressing mutant Tau.

## Results

### System for the rapid generation of human cell models expressing protein-of-interest

**Figure 1:**
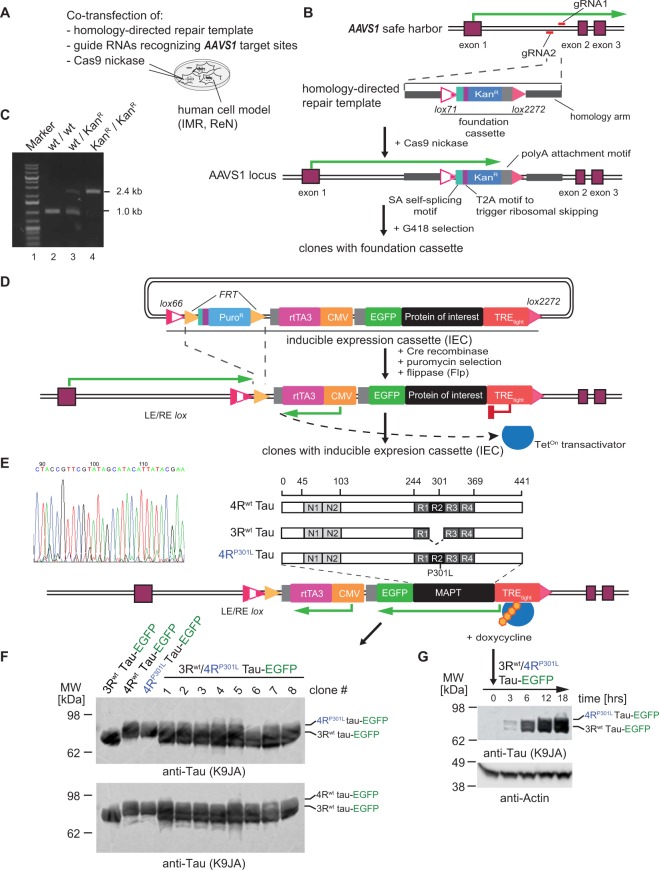
Generation of human cell models for inducible and traceable Tau analysis. (**A**) Schematic summarizing co-transfection step used to generate foundation cassette (FC) in *AAVS1*. (**B**) CRISPR/Cas9 nickase-based intron 1-directed integration of FC within the human safe harbor *AAVS1* locus. The step was based on the homology-directed precise insertion of a repair template featuring a kanamycin-resistance (Kan^R^) gene, flanked by suitable lox acceptor sites. (**C**) Agarose gel depicting genotype analysis of selected clones from three different cell lines that were wild-type, heterozygote or homozygote with respect to the genome-edited *AAVS1* locus. (**D**) Cloning of doxycycline-responsive inducible expression cassette (IEC) flanked by *lox* sites compatible with the corresponding *lox* sites present in the integrated FC at the *AAVS1* locus. Cre recombinase was used to swap FC and IEC within the *AAVS1* locus. The heterologous lox sequence exchange by Cre resulted in doubly mutated LE/RE *lox* sites that resist reversion of this genome-editing step. The kanamycin resistance module was removed by flippase-mediated site-directed recombination of flippase recognition target (*FRT*) sites. (**E**) Design of inducible expression cassettes coding for 4R^wt^, 3R^wt^ or 4R^P301L^ Tau, C-terminally fused to EGFP. The left panel shows genomic Sanger sequencing data covering a transition of wild-type and heterologous sequences within the *AAVS1* locus, thereby validating the successful insertion. (**F**) Tau expression screen of clones, which were designed to express only one Tau isoform or a combination of 3R and 4R Tau isoforms, following Cre recombinase-mediated exchange of Tau expression cassettes into parent cells that were heterozygote or homozygote for the *AAVS1*-targeted FC. Doxycycline induction proceeded for 12 hours before cells were lysed and analyzed by western blotting with the anti-Tau antibody. (**G**) Time-course expression analysis of 3R^wt^ and 4R^P301L^ Tau from the two *AAVS1* alleles present in a successfully genome-edited IMR clone after being treated with 2.0 µg/mL doxycycline for various lengths of time (see Supplementary Fig. S4 for raw data of western blot panels shown in this figure).

To achieve reliability and speed, parental neuroblastoma cells were genetically engineered in two steps. First, a foundation cassette (FC), comprising an antibiotic resistance marker against G418, flanked by specific lox sites, was inserted by CRISPR-Cas9 into both alleles of the previously validated *AAVS1* genomic safe harbour^20^ (Fig. 1A). This step was accomplished by directing a plasmid-encoded CRISPR-Cas9 nickase with a pair of guide RNAs (see Supplementary Fig. S1) to the first intron of the *AAVS1* locus to generate a staggered cut. To facilitate FC integration during the repair of this cut by the high-fidelity, cell-autonomous homology-directed recombination program, the co-transfected FC was flanked by ~1 kilobase pair long homology arms matching either side of the *AAVS1* integration site (Fig. 1B)^21^. Subsequent G418 selection of FC-positive clones was followed by genomic PCR-based amplification and sequencing of *AAVS1* integration sites (Fig. 1C). With these parent clones in hand, the production of specific cell clones expressing any protein-of-interest can be initiated by the insertion of the protein’s coding sequence into a specific cloning site within an inducible expression cassette (IEC) on a customized plasmid. Additional features within the IEC code for the expression of a puromycin resistance marker (Puro^R^) and a version of the Tet^On^ reverse transactivator (rtTA_3_) that exhibits exquisite sensitivity to doxycycline^22^. All modules within the IEC were flanked by lox sites that are compatible to those present in the FC (Fig. 1D). Co-transfection of the plasmid and Cre recombinase into FC-positive parent clones triggered a unidirectional and irreversible (due to the choice of specific lox sites that flank the two cassettes) swap-in of IECs. Subsequent isolation of positive clones relied on puromycin selection. As has been established in previous studies^23^, the EGFP tag fulfils a dual role by enabling traceability of fusion proteins-of-interest based on the fluorescence it emits and by serving as a ligand for the affinity capture of fusion proteins and their interactors^24^. The similar sizes of the EGFP ligand and the GFP binding protein (GBP) bait are favourable for achieving a high density of ligand-bait pairings.

### Validation of inducible Tau models

The utility of the system was tested by generating human cell models that can be induced to express specific Tau isoforms fused to EGFP. More specifically, human 3R and 4R wild-type or P301L mutant Tau sequences were cloned immediately 5-prime to the EGFP coding sequence into the IEC and swapped into the *AAVS1* locus harboring the foundation cassette as described above (Fig. 1E). This step led to the generation of IMR derivative clones that expressed specific isoforms of 3R wild-type, 4R wild-type, or 4R P301L mutant Tau, or combinations of these isoforms from the two gene-edited *AAVS1* alleles available in this diploid cell model (Fig. 1F). For subsequent experiments, we sought to determine how the P301L mutation affects Tau interactions in cells that could be induced to express balanced ratios of 3R^wt^ and 4R^P301L^ isoforms from the two *AAVS1* alleles, with 3R^wt^/4R^wt^ Tau-EGFP expressing cells serving as the reference. Pilot time-course experiments, which monitored cellular 3R^wt^/4R^P301L^ Tau expression levels at suitable intervals, documented no promoter leakage in the absence of doxycycline and established that the addition of 2 μg/ml of this inducer to the cell culture medium caused heterologous Tau protein to be detectable by western blot analysis after 3 hrs (Fig. 1G), with levels increasing further until 18 hrs post-induction (and plateauing thereafter).

### Quantitative Tau interactome analyses

**Figure 2:**
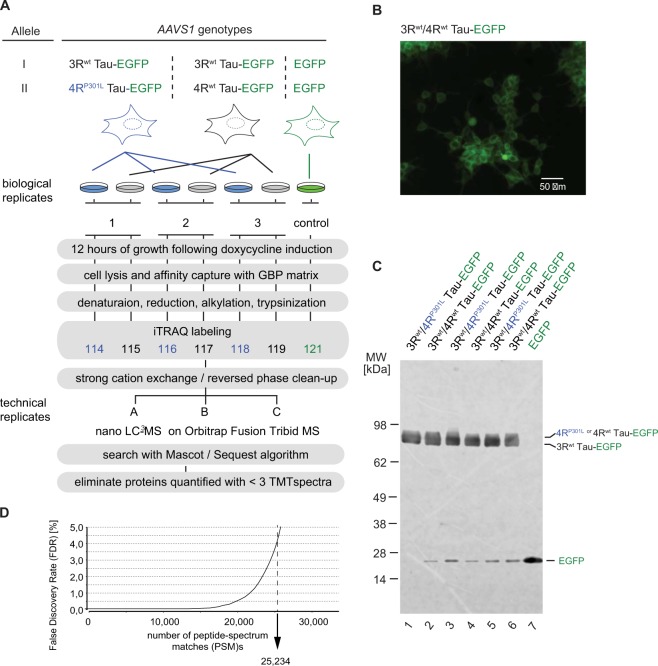
Tau interactome analysis. (**A**) Workflow of comparative interactome analysis of 3R^wt^/4R^wt^ Tau versus 3R^wt^/4R^P301L^ mutant Tau. All proteins of interest were C-terminally fused to EGFP. Analogously generated cell clones expressing EGFP served as the negative control. 12 hours following doxycycline addition to the cell culture medium, cells were lysed and protein complexes comprising Tau-EGFP fusions or EGFP alone were captured on beads saturated with GFP binding protein (GBP). Following extensive washing of beads, affinity-captured proteins were eluted by rapid acidification, denatured in the presence of 6 M urea, reduced with tris(2-carboxyethyl)phosphine (TCEP), alkylated with 4-vinyl pyridine (4-VP), and trypsinized. Next, the peptide mixture was isobarically iTRAQ-labeled and analyzed by mass spectrometry. (**B**) Representative live-cell immunofluorescence analysis of IMR cells expressing 3R^wt^/4R^P301L^ Tau-EGFP 12 hours after induction by addition of 2 µM doxycycline to the cell culture medium. (**C**) Western blot analysis of equal volumes (2.5% of total volume) of affinity-capture eluate fractions. Note that protein levels were comparable in all fractions, and at 12 hours following induction there was no evidence for Tau degradation, except for a minor endoproteolytic cleavage product whose apparent molecular weight is consistent with a partial release of EGFP from the Tau-EGFP fusion. (**D**) Graph depicting depth of interactome analysis on the basis of the number of peptide-to-spectrum matches (PSMs) that passed a 4.4% false discovery rate (FDR) threshold.

For subsequent interactome analyses undertaken in this study, Tau expression was induced by doxycycline addition to the cell culture medium 12 hrs before cell harvest. The sample work-up scheme followed a previously optimized protocol for the identification of Tau binders (Fig. 2A)^13^. As expected, the induction of the Tau-EGFP fusions could be monitored by fluorescence microscopy (Fig. 2B) but no apparent differences were observed in the subcellular distribution of heterologous wild-type or P301L Tau fusion proteins (not shown). Cells were lysed and Tau-EGFP baits were immunoprecipitated on GFP binding protein (GBP) matrices. The capture of fusion proteins could be monitored in real-time on account of the fluorescence emitted by the bait and was accomplished within 2 h. Interactome analyses of 3R^wt^/4R^P301L^ Tau-EGFP samples, and 3R^wt^/4R^wt^ Tau-EGFP controls were undertaken side-by-side in triplicate. To detect non-specific binders to the affinity matrix and to EGFP, an additional control produced identically as the Tau-EGFP fusion clones but expressing only the EGFP fluorescent marker was derived from IMR cells (Fig. 2C). To facilitate direct comparisons of samples and controls and to minimize run-to-run variance amongst replicates, a multi-plex strategy that relied on the isobaric labelling of tryptic peptide mixtures using iTRAQ tags was employed. The tandem mass spectrometry analyses of the pooled and iTRAQ-labelled peptide mixtures were undertaken on an Orbitrap Fusion Tribrid instrument and produced 25,234 peptide-to-spectrum matches (PSMs), which passed a 4.4% false discovery rate (FDR) (Fig. 2D).

### The Tau interactome in IMR cells

**Figure 3:**
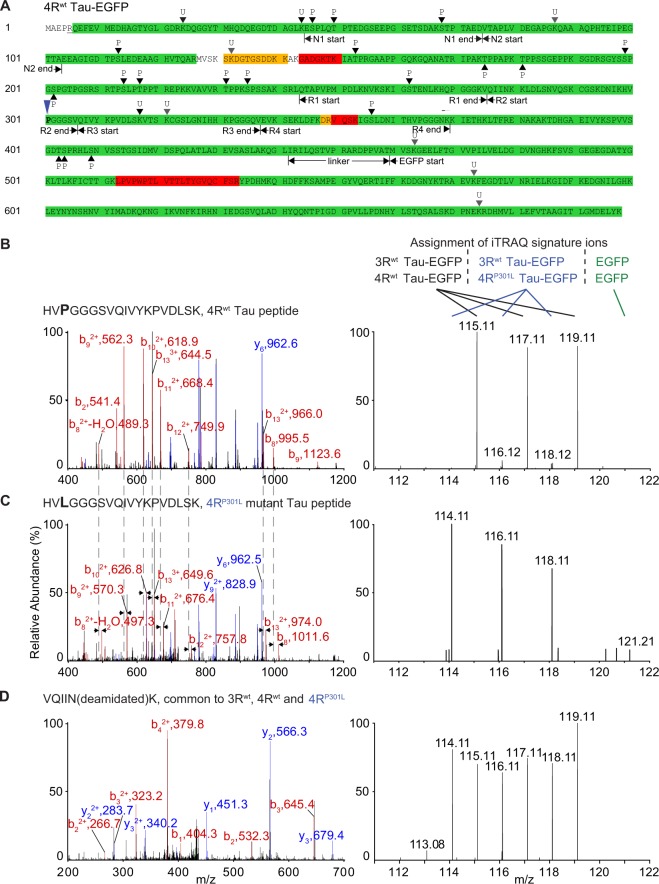
Tau sequence coverage, post-translational modifications and validation of wild-type and P301L mutant Tau sample assignments. (**A**) Mass spectrometry analysis led to highly confident assignments of PSMs that cumulatively covered more than 97% of the amino acid sequence of 4R Tau-EGFP (see also Supplementary Fig. S2). Green and yellow background shading designates segments of the protein that were identified on the basis of PSMs whose assignments exceeded 99% and 95% confidence thresholds, respectively. Assignments with confidence levels below 95% are depicted with red shading. Phosphorylation sites identified by both PEAKS and Proteome Discoverer were labeled with ‘P’. The letter ‘U’ designates evidence for ubiquitination identified on the basis of the characteristic glycine-glycine (GlyGly) stem left behind when ubiquitin is trypsin-digested. GlyGly modifications identified by both search engines or just one of them are marked with black and grey triangles, respectively. (**B**) Left panel: MS2 spectrum assigned to a peptide within 4R Tau that comprises residue P301 and, therefore, should only be contributed by affinity-capture eluates derived from cells expressing 4R^wt^ but not 4R^P301L^ Tau. Right panel: Linked MS3 spectrum generated by fragmenting the ten most intense ions seen in the MS2 spectrum to the left. Note that the intensity profile of iTRAQ signature ions exhibited the expected distribution, thereby validating the integrity and intended order of samples. (**C**) This panel depicts the corresponding MS2 and MS3 spectra obtained by fragmenting the 4R Tau peptide comprising the P301L mutant. As expected on the basis of the iTRAQ labeling scheme, the MS3 spectrum is characterized by an iTRAQ signature ion profile that is complementary to the one seen above in panel ‘A’. (**D**) Peptide-to-spectrum match and its derived MS3 spectrum mapped to a region present in both 3 R and 4 R Tau isoforms.

The computational analysis of this dataset against the human UniProt sequence database revealed Tau to be the strongest hit, with more than 3,817 peptide-to-spectrum matches assigned to it and more than 90% of its amino acid sequence covered by high confidence PSMs (Fig. 3A). MS2 spectra interpreted to encompass Tau residue 301 were characterized by low mass iTRAQ reporter ions observed in MS3 whose relative intensities reflected the isobaric labelling scheme (Fig. 1A), thereby providing a valuable intrinsic control for validating that the CRISPR-Cas9 cloning proceeded as intended and cell clones expressing wild-type Tau (Fig. 3B) or P301L mutant Tau (Fig. 3C) were indeed assigned to iTRAQ labels as planned. Conversely, Tau-derived peptides, which did not encompass amino acid 301, gave rise to MS3 windows characterized by iTRAQ reporter ions of relatively even intensity, which validated that similar amounts of the Tau-EGFP fusion protein bait had been captured from wild-type and P301lL mutant Tau lysates (Fig. 3D). A deeper analysis of Tau-derived mass spectra (Supplementary Fig. S2) based on the PEAKS algorithm revealed, aside from widespread iTRAQ labelling of peptides, many phosphorylation events (see below for details).

**Figure 4:**
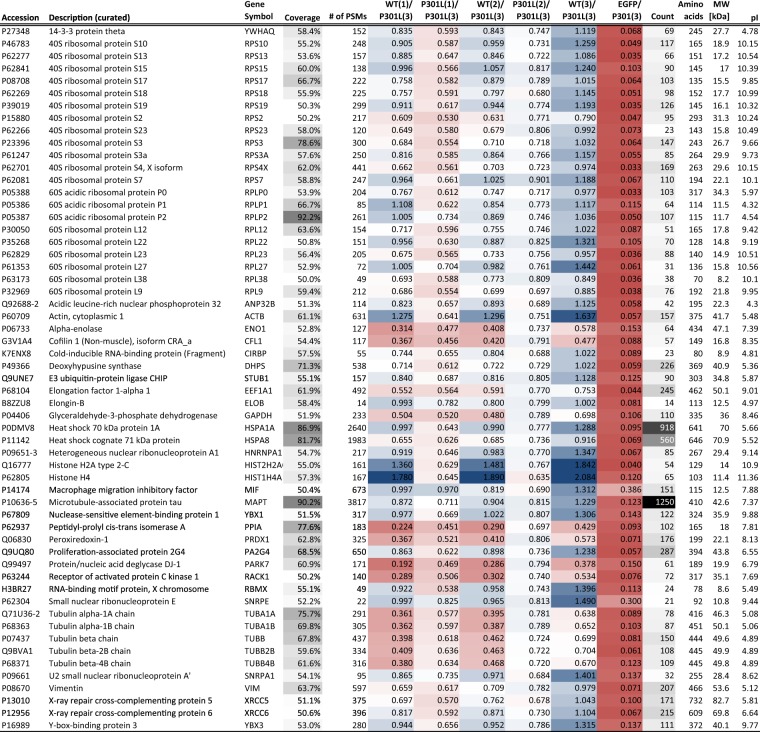
Tau interactome in IMR cells (subset restricted to entries that exceeded 50% sequence coverage; sorted alphabetically). Grey shading was used in this figure to emphasize relative high numbers in the ‘Coverage’ and ‘Count’ columns that depict the protein-specific sequence coverages and numbers of quantified peptide-to-spectrum matches, respectively. Blue-to-red conditional formatting (percentile-based) was used to depict relative iTRAQ signature ion abundance ratios, with the iTRAQ 119 channel intensity, derived from one of three 3R^wt^/4R^P301L^ Tau-EGFP derived biological replicates, serving as the denominator.

In all, the IMR-based Tau interactome dataset comprised 299 proteins. 57 of them were identified based on more than 50% sequence coverage (Fig. 4). Prior annotations available for these proteins indicated a strong Tau-dependent co-enrichment of heat shock proteins and subunits of ribosomal complexes (tallying 68 proteins in the dataset) (see Supplementary Fig. S3 for full list). Other conspicuous groups of Tau interactors observed in the dataset were 14-3-3 proteins, eukaryotic translation initiation factors, heterogeneous nuclear ribonucleoproteins, several RNA binding proteins (FUS, EWS), members of the T-complex, and tubulins. Taken together, this analysis validated the notion that Tau associates in this neuroblastoma cell model primarily with the ribonucleoproteome^13^.

### Selective co-enrichment of DJ-1 with P301L mutant Tau

**Figure 5:**
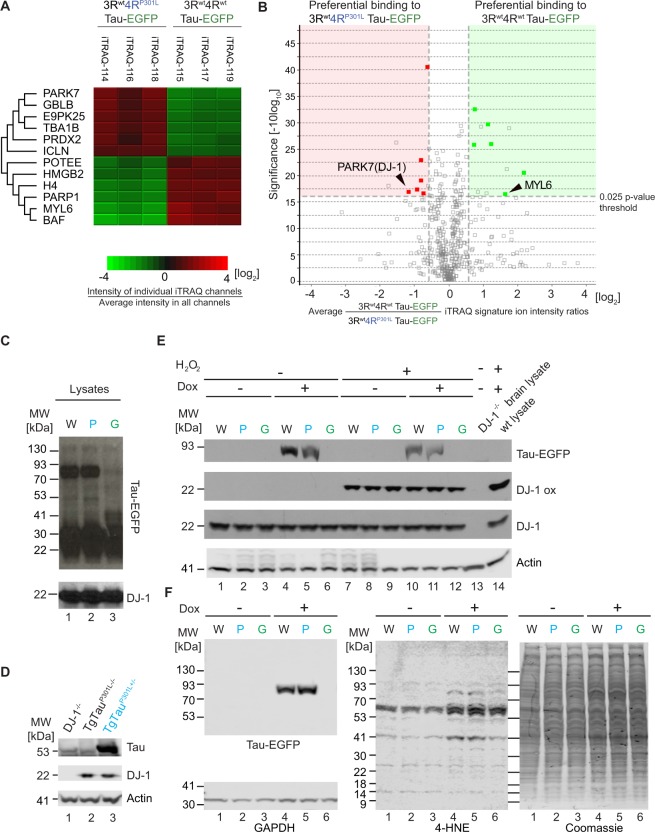
Preferential binding of DJ-1 to mutant 4R^P301L^ Tau, relative to 4R^wt^ Tau, in IMR cells. (**A**) Hierarchical clustering analysis of proteins that exhibited preferential binding to 4R^P301L^ or 4R^wt^ Tau. (**B**) Orthogonal confirmation of proteins shortlisted in panel ‘A’ by volcano plot (ANOVA, *p* = 0.025). Red and green background shading signify preferential binding to 3R^wt^/4R^P301L Tau^ and 3R^wt^/4R^wt^ Tau, respectively (fold change greater than 50%). (**C**) Western blot documenting that the 12 hours induction of wild-type or P301L mutant Tau-EGFP fusion proteins did not alter steady-state levels of DJ-1. Note that in this and subsequent western blot panels samples derived from wild-type human Tau-EGFP expressing cells, P301L mutant Tau-EGFP expressing cells, and EGFP expressing cells are labeled as ‘W in black font’, ‘P’ in blue font and ‘G’ in green font, respectively. (**D**) Similarly, transgenic overexpression of P301L mutant Tau had no apparent effect on DJ-1 expression levels in 2-year-old mice. Specificity of the anti-DJ-1 antibody was validated with age-matched brain extracts from a DJ-1^−/−^ mouse. (**E**) Hydrogen peroxide (H_2_O_2_) treated IMR cells expressing wild-type or P301L mutant Tau-EGFP fusion proteins exhibited no differences in total DJ-1 levels or of its oxidation at residue C106. As negative and positive controls for oxidized DJ-1 served brain lysates from DJ-1^−/−^ and wild-type mice, respectively, that were subjected to 1 mM H_2_O_2_ for 10 min at room temperature. (**F**) No differences in 4-hydroxy-trans-2-nonenal (4-HNE) levels were observed in IMR cells 12 hrs after induction of wild-type versus P301L mutant Tau. Note that although the expression of the Tau-EGFP fusion proteins altered the bands recognized by the 4-HNE-directed antibody, these signals were not different in cells expressing wild-type versus P301L mutant Tau-EGFP fusions (see Supplementary Fig. S5 for raw data of western blot panels shown in this figure).

Because the key objective of this work was to advance our understanding of how P301L mutant Tau might perturb cellular biology causing toxicity, the focus of downstream analyses was on proteins that exhibited significantly (p < 0.025) differential binding to P301L mutant Tau. Hierarchical clustering of median iTRAQ reporter ion intensities for all 299 candidate Tau interactors identified several proteins that fulfilled this criterion (Fig. 5A). Amongst these, PARK7, also known as the human protein deglycase DJ-1, stood out by its most selective co-enrichment with P301L mutant Tau (Fig. 5B). Mindful of the P301L disease phenotype being characterized as a frontotemporal atrophy with degeneration of basal ganglia that involves the substantia nigra^25^, DJ-1 also caught our attention by its known role as a gene whose mutations cause recessive forms of Parkinson’s disease. In subsequent validation experiments, we learned that the preferential DJ-1 co-enrichment with P301L mutant Tau in this cell model does not merely reflect trivial differences in steady-state DJ-1 protein levels, because the doxycycline-based induction of wild-type and P301L mutant Tau did not affect DJ-1 protein levels in IMR cells (Fig. 5C). Similarly, chronic overexpression of P301L mutant Tau in a previously reported transgenic mouse model had no effect on steady-state DJ-1 levels. Moreover, no changes to endogenous Tau levels were observed in DJ-1-deficient mice (Fig. 5D). DJ-1 is widely considered to act as a redox-sensitive chaperone and to undergo oxidation of its cysteine residue 106 in response to oxidative stressors *in vitro*^26,27^ and *in vivo*^28^. Considering that P301L mutant Tau may perturb this aspect of DJ-1 biology, we next exposed IMR cells expressing wild-type or P301L mutant Tau-EGFP to hydrogen peroxide treatment and monitored total DJ-1 levels as well as the oxidation of DJ-1 at its residue C106 by western blotting (Fig. 5E). This approach led to the expected C106 oxidation in hydrogen peroxide-treated cells but did not reveal an influence of wild-type or P301L mutant Tau on the oxidation of this critical DJ-1 cysteine residue. We therefore turned to an alternative method of assessing if P301L mutant Tau may influence overall redox stress, namely by monitoring levels of lipid peroxidation. Because of its reactivity toward proteins, the detection of 4-hydroxynonenal (4-HNE), a prominent lipid peroxidation product, can serve as a surrogate for oxidative stress levels and was targeted here to assess the effect of P301L mutant Tau on cellular redox stress levels. This experiment revealed that 4-HNE adducts were indeed more readily detected in cells induced to express Tau-EGFP constructs (Fig. 5F). However, no differences in the levels of prominent 4-HNE adducts were observed in cells 18 hrs after induction of wild-type or P301L mutant Tau. Taken together, this line of experimentation, although intriguing due to the shared connection of P301L mutant Tau and DJ-1 mutations to substantia nigra degeneration, has so far not revealed a clear indication that the preferential P301L mutant Tau binding to DJ-1 translates into a difference in DJ-1-related redox stress levels.

### Tau interactome analysis in differentiated co-cultures of neurons and astrocytes

**Figure 6:**
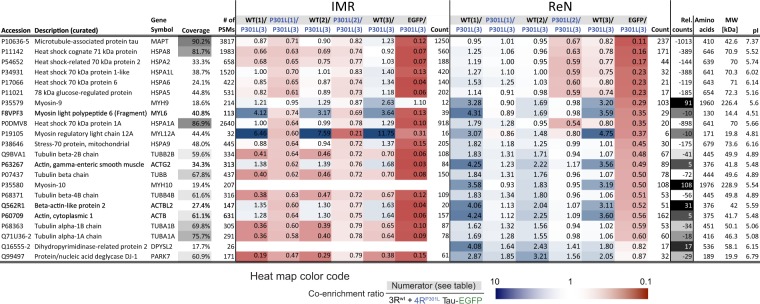
Comparison of Tau interactomes in IMR and ReN cells. Entries sorted by enrichment levels relative to EGFP-only negative controls observed in ReN cells. Please see also legend of Fig. 4 for a description of grey-shades and color-shading used in this figure. The relative counts (Rel. counts) column depicts the protein-specific difference count of peptide-to-spectrum matches observed in IMR and ReN cells. Note the relatively high difference counts for non-muscle myosin-9 and non-muscle myosin-10, reflecting the pronounced co-enrichment of these proteins with Tau in ReN cells.

An obvious limitation of analyses up to this point was the reliance on a system that explored Tau interactions in dividing cells. Because mitotically active cells must allocate considerable resources to the cell division program, the concern arises whether and how the signalling pathways that underpin morphogenetic changes in proliferating cells influence other aspects of cell biology. In regard to Tau, there is the additional caveat that human brain Tau levels are observed to be highest in axons of differentiated neurons, a subcellular compartment that cannot be studied in the IMR cell model. To address this concern, in a parallel strand of investigation, the CRISPR-Cas9-based genetic engineering steps described for the IMR cell line (Fig. 1) were reproduced in ReN cells. The latter represent a mixed-population human neuroprogenitor cell line that can be differentiated into co-cultures of glial cells and neurons. Using an enhanced differentiation scheme (hereafter termed pre-aggregation differentiation, or PreD) that relies on culturing the cells as neurospheres first and later supplementing the differentiation medium with glial-derived neurotrophic factor and dibutyryl-cyclic-adenosine monophosphate, ReN cell neurons have been shown to form fully electrochemically active axons that can be induced to elicit action potentials^29^. As described in detail for the IMR cell model, we were able to generate doxycycline-inducible wild-type and P301L mutant Tau-EGFP-expressing *AAVS1* knock-in ReN cell derivates, as well as the corresponding EGFP-expressing negative controls. However, because ReN cells predominantly express 3R Tau endogenously^30^, we chose to not insert an additional 3R Tau expression cassette into the second *AAVS1* allele to achieve a near-balanced 3R/4R Tau ratio. Next, we differentiated these lines, borrowing from previously published procedures^19,29^, induced 4R Tau expression for 12 hrs by doxycycline addition and captured the bait proteins on GBP matrices, as we had done before (Fig. 2A). In order to increase stringency, i.e., identify only proteins whose Tau binding can tolerate high levels of salt, affinity matrices were this time subjected to two consecutive rinses with buffers that contained 500 mM NaCl. The subsequent mass spectrometry analyses followed procedures established in the IMR-based interactome analyses. This more stringent approach led again to the highly specific capture of Tau but resulted in the identification of a smaller number of Tau candidate interactors (Fig. 6, see also Fig. S3). A characteristic difference of this ReN cell-based Tau interactome dataset was a conspicuous absence of proteins belonging to the cellular ribonucleoproteome, which had dominated the IMR cell-derived Tau interactome dataset. Consistent with the IMR results, Tau was again observed to primarily interact with heat shock proteins, irrespective of whether the bait comprised wild-type or P301L mutant Tau. The ReN cell-based analyses also validated DJ-1 as a robust Tau interactor but, remarkably, and in contrast to the IMR-based data, indicated a preference of DJ-1 to interact with wild-type Tau, rather than P301L mutant Tau, in differentiated co-cultures of neurons and astrocytes (Fig. 6).

### Preferential binding of wild-type Tau to non-muscle myosins

**Figure 7:**
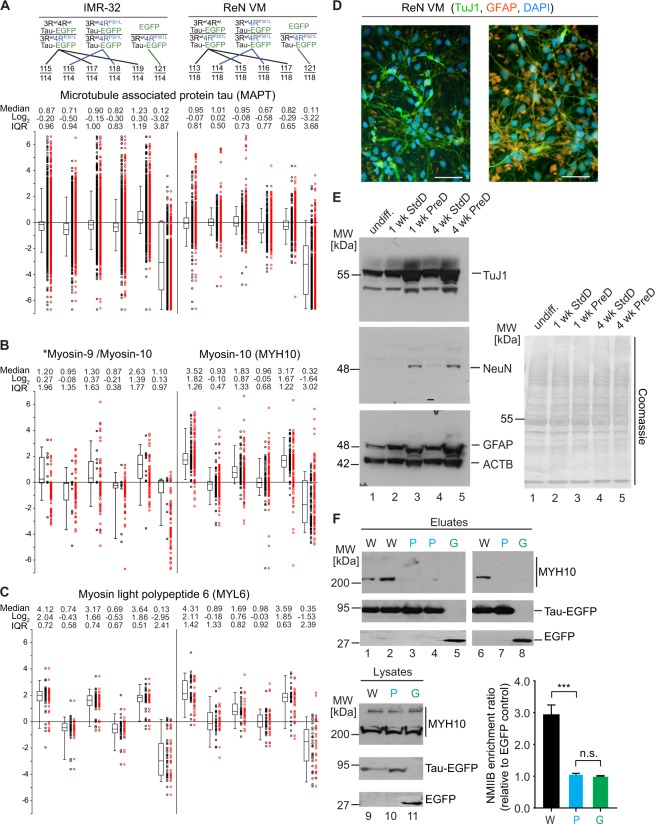
Preferential binding of wild-type Tau to non-muscle myosins. (**A**) Box plots adapted from ProteomeDiscoverer software depicting abundance ratios of peptides assigned to Tau in IMR and ReN cell interactome datasets. In this type of graph, ratios of iTRAQ reporter ion intensities for a given peptide are depicted as individual dots. Because the data were queried with Mascot and Sequest algorithms, most PSMs were assigned to Tau by both algorithms. To avoid artificial inflation of hits, only unique and confident assignments were considered when computing median ratios and inter quartile ranges (IQR), i.e., duplicate or low confidence PSMs (red dots) were ignored. As expected, Tau levels were similar in all eluate fractions except for negative controls. (**B**) Box plots for non-muscle myosin-10 (MYH10) revealed its preferential enrichment in wild-type Tau co-immunoprecipitates. *Whereas peptide-to-spectrum matches in the ReN cell-derived Tau interactome dataset unequivocally identified MYH10, the IMR cell-derived dataset comprised only a small number peptide-to-spectrum matches for this protein that were shared with its paralog MYH9. (**C**) Box plots results for myosin light polypeptide (MYL6). (**D**) Distribution of TuJ1-positive neuronal cells and GFAP-positive glial cells in two fields of vision that were mostly composed of neuronal (left panel) and glial cells (right panel) after one week of PreD differentiation. TuJ1: green, GFAP: red, DAPI: blue. Scale bar: 50 μm. (**E**) Preaggregation method of differentiation (PreD) increases expression of neuronal markers more robustly than the standard method of differentiation (StdD). Western blot analyses of ReN VM cell lysates before and after one week or four weeks of differentiation using the StdD or PreD method. Expression of neuronal marker TuJ1 is appreciably higher in PreD-differentiated cells than in StdD. Expression of neuronal marker NeuN is present only in PreD-differentiated cells. Whereas expression levels of TuJ1 and NeuN were similar in cultures that were differentiated 1 week or 4 weeks, GFAP signal intensities continued to increase in PreD differentiated cultures from 1 to 4 weeks of culture. (**F**) Validation of selective co-enrichment of MYH10 with wild-type Tau. Western blots depicting co-immunoprecipitation eluate fractions and input lysates derived from cells expressing wild-type Tau-EGFP (labeled as ‘W’), P301L Tau-EGFP (labeled ‘P’ in blue font) or EGFP (labeled as ‘G’ in green font). Lower right panel: quantitation of signal intensities of western blot bands. Values were computed from triplicate analyses. All data are represented as mean ± SEM (see Supplementary Fig. S6 for raw data of western blot panels shown in this figure).

Presumably at least in part reflecting the increase in stringency, the levels of Tau itself and most of its candidate interactors were reduced (apparent by the reduction in total PSMs observed) in the ReN cell-based Tau interactome dataset, relative to the IMR-based analyses, which had been undertaken at the same scale (Fig. 7A). The most striking exception to this overall trend was represented by non-muscle myosins, which were identified with their heavy and light chains and were detected in the ReN cell Tau interactome dataset with a considerable proportion of total PSMs captured (Fig. 6). The latter not only was reflected in improved statistics of their iTRAQ reporter ion profiles but also brought to the fore a profound preference of their binding to wild-type Tau, relative to P301L mutant Tau. Although the same trend had existed in the IMR dataset, its robustness in the ReN cell data triggered further interest in this interaction (Fig. 7B,C).

In validation work, we verified the previously described increase in the expression of neuronal markers, neuronal nuclear antigen (NeuN) and Class III β-tubulin (TuJ1)^29^, but also observed a parallel boost to the expression of glial fibrillary acid protein (GFAP) in cells that had undergone astrocytic differentiation (Fig. 7D,E). These changes in the expression of neuronal and astrocytic markers was already apparent 1 week into the PreD differentiation. Beyond one week of differentiation open spaces left in the cell culture dishes subject to the PreD protocol predominantly filled with astrocytes, evident by a marked increase in the GFAP signal, but not TuJ1 or NeuN signals, in 4-week PreD treated cultures. This observation corroborated our choice to work with 1 week differentiated ReN cells for the interactome analyses, when neurons represented approximately 20% of the total cell population on the basis of cell counts of TuJ1- versus GFAP-positive cells in representative fields of vision. Subsequent western blot analyses of GBP-affinity capture eluates validated the selective co-enrichment of non-muscle myosin with wild-type Tau, which easily passed significance thresholds (p < 0.001). This preferential association could not be explained by differences in the expression levels of bait proteins or MYH10 in lysates, which were not observed (Fig. 7E).

**Figure 8:**
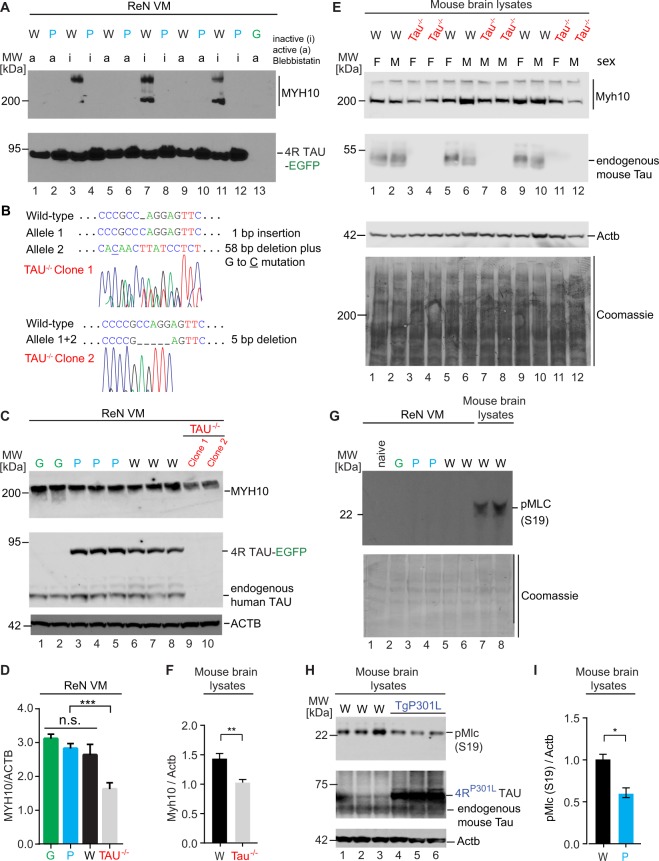
Tau binding to MYH10 is inhibited by blebbistatin and may stabilize steady-state levels of MYH10. (**A**) Binding of wild-type Tau to MYH10 is blocked when non-muscle myosin II ATPase activity is inhibited by treatment with 10 µM of the active (S)-Blebbistatin enantiomer for 2 hours. Western blot analysis documenting that MYH10 binding to wild-type Tau-EGFP can be blocked by active (a) but not by inactive (i) blebbistatin, a cell-permeable inhibitor of non-muscle myosin II ATPases. (**B**) Genetic sequencing results of TAU^−/−^ cell clones (sequencing chromatogram retraced to enhance signal strength). The position of indels was inferred from the partial sequence alignment to the wild-type *MAPT* sequence. (**C**) The presence or absence (TAU^−/−^) of endogenous Tau influences levels of MYH10 in 1-week differentiated ReN cells. (**D**) MYH10 levels in TAU^−/−^ cells were significantly lower (***p < 0.001) than in inducible Tau-EGFP or EGFP expressing cells. MYH10 levels were normalized to β-actin (ACTB) levels. (**E**) Comparison of Myh10, endogenous Tau and Actb levels by western blot analysis in mouse brain extracts of wild-type and Tau^−/−^ mice. The lower panel depicts a portion of the Coomassie-stained western blot to document equal total protein loading. (**F**) MYH10 protein levels were significantly (p < 0.01) lower in Tau^−/−^ mice than in age-matched wild-type C57BL/6 control mice. (**G**) Attempts to undertake a phospho-MLC (pS19) analysis with ReN cell extracts have failed to date because the respective antibody only reacts with mouse samples, not human samples. Naïve ReN VM cells are shown in lane 1 to control for clonal effects of inducible cells. A portion of the Coomassie blue-stained western blot validates equal protein loading. (**H**) Transgenic P301L Tau mice exhibit reduced levels of phospho-occupancy at residue S19 within their regulatory myosin light chains. (**I**) Bar graph: quantitation of signal intensities of pS19 western blot bands derived from triplicate analysis of brain lysates of P301L transgenic mice versus control mice of same genetic background. All data are represented as mean ± SEM. For sample abbreviations, please see previous legend (see Supplementary Fig. S7 for raw data of western blot panels shown in this figure).

The biology of non-muscle myosins is in large part governed by ATP catalysis within the head domain of the heavy MYH10 subunit. To assess if the interaction of wild-type Tau with MYH10 is responsive to changes in ATP turnover, we next exposed differentiated ReN cells to blebbistatin, a reagent known to specifically inhibit the ATPase function of non-muscle myosins. As negative controls, cells were treated side-by-side with an inactive blebbistatin enantiomer. Strikingly, this experiment revealed that binding of wild-type Tau to MYH10 requires an uninhibited ATPase activity, as binding was abolished in the presence of active blebbistatin (a) but not its inactive enantiomer (i) (Fig. 8A).

Frequently, interactions amongst proteins shields them from degradation. Conversely, when a protein is missing, its interaction partners are often observed at lower steady-state levels. To investigate if this scenario applies to the Tau-MYH10 interaction, *MAPT*-specific paired single-guide RNAs and a Cas9 nickase expression cassette were introduced into ReN VM cells to achieve a coding sequence shift that triggers nonsense mediated decay and ablation of endogenous Tau expression. Two distinct *MAPT*^−/−^ clones obtained were analysed by western blotting for their expression of MYH10. This experiment showed that MYH10 levels were indeed significantly reduced in the absence of endogenous Tau (Fig. 8B–D).

We next assessed if the aforementioned differences in binding of wild-type versus P301L Tau to non-muscle myosins manifest in differences in the activity of the latter *in vivo*. To this end, we compared TgP301L mice with non-transgenic control mice of identical genetic background and capitalized on the fact that the phosphorylation status at amino acid Serine 19 of the regulatory myosin light chains (Mlc), including the MYL6 we found to interact with wild-type Tau, is known to reflect the activity of non-muscle myosin motors. Consistent with the notion that binding of wild-type Tau to non-muscle myosins may stabilize the activity of non-muscle myosins, transgenic mice expressing P301L exhibited a significant reduction in the phosphorylation status of Mlc (Fig. 8E–G).

### Wild-type and P301L Tau are not differentially phosphorylated 12 hours upon induction

**Figure 9:**
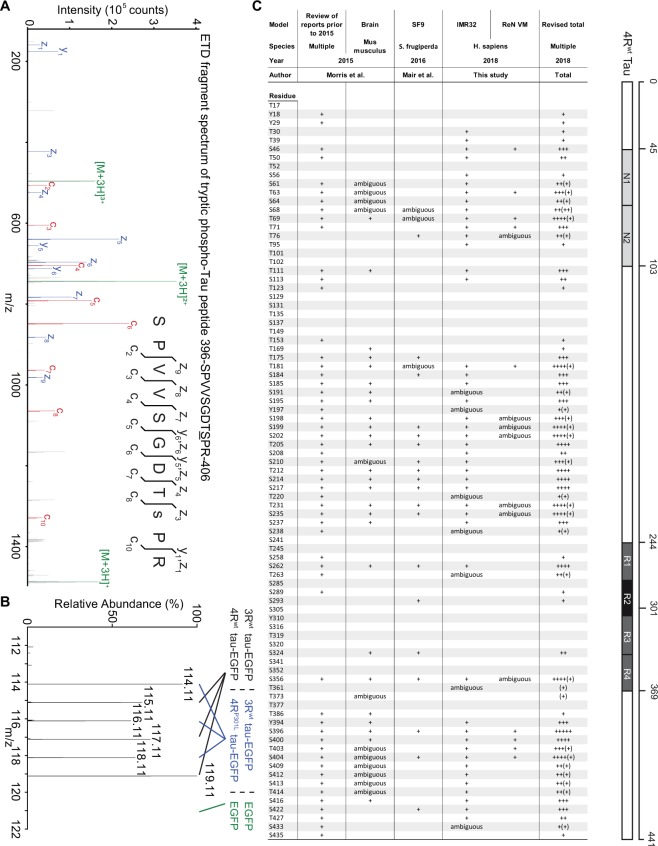
Wild-type and P301L Tau are not differentially phosphorylated - inventory of Tau phosphorylation sites. (**A**) Representative electron transfer dissociation (ETD) spectrum, depicting a highly confident identification of Tau phosphorylation at its serine residue 404, revealed by fragmentation of the tryptic peptide 396-SPVVSGDT**S**PR-406. (**B**) MS3 spectrum linked to ETD fragment spectrum shown in panel ‘A’. Note that levels of phospho-occupancy were similar in all six iTRAQ reporter ion channels for this phosphopeptide derived from wt or P301L Tau-containing co-immunoprecipitations. As expected, the 121.1 reporter ion corresponding to the EGFP negative control sample did not show any signal in this spectrum. (**C**) Comparison of Tau phosphorylation sites identified in prior reports and this study. The column, which lists assignments made in this study depicts only the most conservative interpretations, i.e., only MS2 spectra were considered whose fragmentation pattern included ions that allowed confident assignments to specific phosphoacceptor sites. Since many tryptic peptides of Tau comprise several candidate phosphoacceptor sites and frequently unique assignments were not possible, the true number of Tau phosphoacceptor sites might be larger (see also Supplementary Fig. S2).

The 441 amino acid human 2N4R Tau protein sequence contains 85 serine, threonine or tyrosine residues. The protein is not only naturally phosphorylated but Tau hyperphosphorylation is a hallmark of AD and has also been observed in P301L mutation carriers. To begin to assess if differences in Tau phosphorylation played a role in the preferential binding of wild-type Tau to non-muscle myosins, iTRAQ-labeled phospho-peptides were enriched on titanium dioxide-matrices and analysed by tandem mass spectrometry, augmented by the complementary use of instrument configurations that generated peptide fragments by collision-induced dissociation or electron transfer dissociation (Fig. 9A). This phase of the project made use of the PEAKS algorithm, followed by visual inspection of mass spectra proposed to harbor phospho-sites. As expected, during 12 h of induction, Tau was observed to be phosphorylated at several sites. More specifically, in IMR cells (the dataset with the highest Tau PTM coverage), more than half of the theoretical phospho-acceptor residues were at least in a subset of spectra interpreted to be phosphorylated (Fig. S2), with assignments to an additional 8 sites considered tentative (Fig. 9B). Spectral count-based estimates (comparing the number of total spectra comprising a given phospho-acceptor site to the number of spectra showing the same site to be phosphorylated) indicated that occupancy rates per site approximated but never exceeded 50% for a few sites. Consistent with earlier data by others, in particular, phospho-acceptor sites N-terminal to the microtubule binding domain and within the C-terminal tail of Tau showed robust phosphorylation, with residues S199, S202, T212, S214, T217, T231, S235, S396, S400, T403, S404 showing the highest occupancy rates. However, 12 hrs following doxycycline-mediated Tau induction, no differences in the phosphorylation patterns or relative occupancies of phosphorylation sites were apparent when comparing iTRAQ reporter ion intensities of Tau peptides derived from cells expressing wild-type or P301L mutant Tau (Fig. 9C).

## Discussion

This study produced and compared two in-depth Tau interactome datasets generated with a dividing and a non-dividing human model, each expressing wild-type or P301L mutant Tau fused to a C-terminal EGFP. Both models corroborated previously reported Tau protein-protein interactions, including its robust binding to heat shock proteins of the Hsp70 protein family, which was observed at similar levels irrespective of the presence or absence of the FTD mutation. It also identified several proteins that were profoundly affected by the mutation in their binding to Tau. Interestingly, whereas for DJ-1 the binding preference to wild-type or P301L mutant Tau presented inconsistently in IMR and ReN cells, non-muscle myosins emerged in this work as preferentially binding to wild-type Tau in both cell models. The Tau binding of non-muscle myosins also stood out by its resilience to harsh washing steps, which caused the removal of a majority of proteins that interact with Tau under physiological salt conditions and are broadly identifiable as members of the ribonucleoproteome.

A conceptual advance of this work was its use of an inducible system for the controlled expression of Tau in a human cell model. The system offers versatility that can also save resources for future IMR or ReN cell-based studies. With the foundation cassettes in the *AAVS1* safe harbor locus in place, and the positive isolation of Cre-mediated integrant clones relying on rapid puromycin resistance selection, the system can be adapted for the expression of other proteins-of-interest in less than a month (a timeline that has been validated in two separate studies – not shown). Here, the doxycycline-responsive inducibility of the system ensured: (1) Tau levels could be titrated by controlling doxycycline levels; and (2) the interactome was not dominated by interactions that govern Tau degradation. Corroborating the latter conclusion, proteins known to be associated with the proteasomal degradation machinery were conspicuously absent from this Tau interactome dataset. This was in stark contrast to a previous Tau interactome dataset we had produced from IMR cells that were stably transfected with a Tau expression plasmid and made use of largely identical workflows and instrumentation. In this previous dataset more than a dozen proteasomal subunits were represented with robust spectral counts (>100) and sequence coverages exceeding 60%^13^.

One recent interactome study investigated binders of Tau in mouse brains^31^. In addition to confirming the ability of Tau to interact with a subset of heat-shock proteins and members of 14-3-3 protein family, the affinity-capture of endogenous mouse Tau led predominantly to the co-enrichment of cytoskeletal proteins and abundant brain proteins, including synaptic proteins and myelin components but not the ribonucleoproteome. The reliance on mouse brains was both a strength and a weakness of this study, the major concerns being that mouse brains may not faithfully recapitulate perturbations in human tauopathies, and the possibility that cell type-specific Tau interactions could have been masked by the complexity of cellular phenotypes present in the brain.

Surprisingly, the current study documented DJ-1 to be preferentially associated with P301L mutant Tau or wild-type Tau in IMR or ReN cells, respectively. This finding serves as yet another data point in an emerging body of literature that cautions against the expectation that protein-protein interactions are mostly conserved when comparisons are made across paradigms^32^. A battery of tests that explored if these observations reflected (1) cell type-specific differences in steady-state expression levels of DJ-1, (2) differences in the cells’ ability to deploy DJ-1 as a scavenger of reactive oxygens, or (3) broader differences in the cells’ coping with redox stress, has to this date not yielded a clue for interpreting this finding (Fig. 5). Despite this shortfall, it is noteworthy that the interaction between DJ-1 and Tau was observed in both paradigms, survived high salt washes of the capture matrix and was one of very few interactions that was quantitatively impacted by the presence of the P301L mutation. In light of the similar Parkinson’s disease-characteristic phenotypes that can be caused by P301L Tau or mutations in DJ-1, further studies into the molecular underpinnings of this link between Tau and DJ-1 may prove to be a productive direction for further research. Various scenarios can be invoked, including the possibility that a specific post-translational modification within Tau or DJ-1 governs this interaction. Relevant insights are expected to emerge from biochemical, immunocytochemical or genetic analyses. The latter could probe for genetic interactions between DJ-1 and Tau and could be based on crosses of mice that are either engineered to harbor the respective mutants or are deficient for one of the parent genes.

Arguably the most striking observation in this work was the preferential and consistent association of non-muscle myosins with wild-type Tau but not its mutant derivative. Not only did this interaction withstand stringent washing steps but the MYH9 and MHY10 heavy chains of non-muscle myosin complexes were the two proteins whose relative spectral counts increased the most when we moved from a workflow that made use of IMR cells and washing of capture matrices under physiological salt conditions to ReN cells and washing of Tau capture matrices under high-salt conditions (tallies of ‘relative counts’ shown in Fig. 6). Follow-up biochemical experiments validated the selective interaction of non-muscle myosins with wild-type Tau (as well as the perturbation of this interaction in P301L mutant Tau capture experiments) and revealed that it relies on active ATPase function in non-muscle myosins. We currently interpret our results to indicate that the interaction of wild-type Tau with non-muscle myosins promotes the normal biology of these cytoskeletal motors and stabilizes their steady-state levels. P301L mutant Tau did not affect the expression of endogenous Tau in the mouse model we tested (Fig. 8E). However, P301L mutant Tau may stand in for endogenous Tau in regard to its binding to microtubules and other Tau-related cellular roles. In this way, the presence of P301L mutant Tau may displace endogenous Tau and partially block its normal interaction with non-muscle myosins, leading to a reduction in the phosphorylation of their light chains.

Non-muscle myosins are best known for providing contractile forces that control a wide range of biological processes, including cytokinesis, phagocytosis, cellular migration^33^ and mitochondrial fission^34^ within non-muscle cells. This subfamily of myosins is also understood to be critical for dendritic spine morphology^35–38^ and has been observed as integral components of the axon initial segment (AIS)^39–41^ and tunneling nanotubes (TNTs)^42^. Tau itself has repeatedly not only been implicated in the cellular biology that governs the aforementioned neuronal structures but the perturbation of its biology in relation to these structures has been proposed to constitute ground zero for the cellular toxicity underlying FTD. Yet, to our knowledge, a direct interaction between Tau and non-muscle myosins has not previously surfaced. However, the unconventional Myosin VI was one of 30 modifier genes of V337M mutant Tau-mediated neurodegeneration in a Drosophila model of FTD^43^. The authors went on to report that Myosin VI associated with Tau tangles in a small number of brains from AD and FTDP-17 cases but not from individuals with a postmortem diagnosis of PSP or Pick’s disease. Several studies have zeroed in on Tau’s relationship to the AIS, mostly motivated by interest in the molecular basis for the predominantly axonal localization that characterizes healthy neurons and is gradually lost in several tauopathies. Intriguingly, both hyperphosphorylated P301L mutant Tau^44^ and activity-impaired non-muscle myosins^40^ have recently been reported in independent studies to displace the AIS further down the axon. Finally, the aforementioned TNT study identified, in addition to actin and MYH10, Tau as a specific marker of TNTs^42^, thereby indicating a subcellular niche where a possible *in vivo* interaction between Tau and a non-muscle myosin might be studied.

Efforts to map the sites of phosphorylation, identify the kinases responsible, and develop an understanding of the relative order and significance of individual phosphorylation sites have marked Tau research for years. Reflective of advances in mass spectrometry instrumentation, the most in-depth analyses of Tau post-translational modifications to date were reported in recent time, including comprehensive phospho-site analyses of mouse brain Tau^45^ and human Tau expressed in SF9 cells^46^. The in-depth Tau sequence coverage obtained in this work afforded yet another opportunity to map phosphorylation sites, with the added advantage that not only the Tau sequence itself but also the host cells were of human origin. Moreover, the concomitant analysis of samples from wild-type versus P301L mutant Tau expressing cells, was used to determine if the P301L mutation enhanced Tau phosphorylation relative to wild-type Tau, and if so, which sites are most affected. These analyses revealed no evidence for P301L-dependent Tau hyperphosphorylation within the 12 hrs induction window and in the two cell models tested. A close look at the literature reporting on the phosphorylation of mutant FTD Tau versus wild-type Tau indicates that this finding is not an outlier. Whereas some FTD mutations, including the R406 mutation, have been reported to cause long-range effects on phosphorylation sites^47^, most analyses of the consequence of expressing P301L mutant Tau in various cell models indicated no or few differences in phospho-site occupancy^48^ and hyperphosphorylation of P301L mutant Tau is typically only observed in adult brain tissue^49^. There are several caveats in the interpretation of data presented in this report that warrant further investigation under separate cover. More specifically, although the most parsimonious interpretation of the current data suggests that preferential binding of non-muscle myosin to wild-type Tau, relative to P301L mutant Tau, did not reflect overall differences in Tau phosphorylation, we cannot comment on whether the sub-pool of Tau that associated with non-muscle myosins was differentially phosphorylated. To firmly address this question a reverse immunoprecipitation followed by in-depth profiling of Tau posttranslational modifications could be informative. Similarly, the current study did not incorporate steps that could delineate the possible contribution of sub-pools of Tau, characterized by distinct subcellular localization or solubility, to differences in wild-type versus P301L mutant Tau interactomes. A more complete account of the underlying differences between wild-type and P301L mutant Tau in their propensity to bind to non-muscle myosins may benefit from detailed insights into how phosphorylation affects solubility. At least *in vitro* there is elegant prior data that documented P301L mutant Tau to exhibit higher aggregation propensity, relative to wild-type Tau, when a lower number of phospho-sites are present^50^.

In the spirit of many interactome studies that came before it, this work raised more questions than it could answer. Amongst the unresolved observations a few stand out: (1) the robust yet inverse co-enrichment DJ-1 exhibited toward wild-type and P301L Tau in the two human cell models, serving as a warning to cautiously interpret results obtained with a specific paradigm but also asking for an explanation. (2) The attention it drew to non-muscle myosins as candidate mediators of a poorly understood biology underlying P301L mutant Tau-dependent toxicity. (3) The fact that differences in the interactions of wild-type and P301L mutant Tau manifested in the absence of apparent differences in their phosphorylation state. Thus, hopefully, a lasting contribution of this report will be that it put a spotlight on a small number of protein-protein interactions that warrant follow-up and provided models that will be useful to anyone interested in pursuing these leads.

## Methods

### Antibodies

Primary antibodies were sourced as follows: rabbit polyclonal K9JA anti-Tau antibody (catalog number A0024, Dako Canada, Burlington, ON; used at 1:2000 dilution), mouse monoclonal JL-8 anti-GFP antibody (catalog number 632380, Clontech Laboratories Inc., Mountain View, CA; used at 1:1000 dilution), rabbit polyclonal anti-DJ-1 antibody (catalog number ab18257, Abcam, Cambridge, MA; used at 1:1000 dilution), rabbit monoclonal MJF-R16 (66-5) anti-oxidized DJ-1 antibody (catalog number ab169520, Abcam; used at 1:500 dilution), rabbit polyclonal anti-HNE reduced Michael adducts antibody (catalog number ABN249, Millipore, Etobicoke, ON; used at 1:1000 dilution), mouse monoclonal 3H2 anti-non-muscle myosin IIB antibody (catalog number ab684, Abcam; used at 1:500 dilution), rabbit monoclonal EPR22564-23 anti-MYH10 antibody (catalog number ab230823, Abcam; used at 1:1000 dilution), mouse monoclonal anti-phospho-myosin regulatory light chain 2 (Ser19) antibody (catalog number 3675, Cell Signaling Technology, Danvers, MA; used at 1:500 dilution), mouse monoclonal anti-β-actin antibody (catalog number AM1021B, Abgent, used at 1:2000 dilution), mouse monoclonal ZG003 anti-GAPDH antibody (catalog number 39–8600, Thermo Fisher Scientific, Nepean, ON; used at 1:2000 dilution), mouse monoclonal TU-20 anti-Tuj1 antibody (catalog number ab7751, Abcam; used at 1:1000 dilution), mouse monoclonal anti-GFAP antibody (catalog number A21282, Life Technologies; used at 1:1000 dilution), rabbit monoclonal EPR12763 anti-NeuN antibody (catalog number ab177487, Abcam; used at 1:1000 dilution), and mouse monoclonal DA9 anti-total tau antibody, a generous gift from Dr. Peter Davis (used at 1:1000 dilution).

Secondary antibodies against mouse (catalog number 7076S; used at 1:10,000 dilution) and rabbit (catalog number 7074S; used at 1:10,000 dilution) immunoglobulin G were purchased from Cell Signaling Technology.

### Experimental models

Human neuroblastoma IMR-32 cells were purchased from the American Type Culture Collection (catalog number CCL-127, ATCC, Manassas, VA, RRID:CVCL_0346). Human neural progenitor ReN VM cells were from MilliporeSigma (catalog number SCC008, MilliporeSigma, Burlington, MA, USA). IMR-32 cells were maintained in DMEM (catalog number 11995-073, Life Technologies, Burlington, ON) supplemented with 10% heat-inactivated fetal bovine serum (catalog number 10082139, Thermo Fischer Scientific), and 1% GlutaMax (catalog number 35050061, Thermo Fischer Scientific) at 37 °C with 5% CO_2_. ReN VM cells were maintained in DMEM/F12 (catalog number 21041025, Gibco) supplemented with 2% N21-MAX (catalog number AR008, R & D Systems, Minneapolis, MN, USA), 20 ng/ml basic fibroblast growth factor (catalog number PHG0261, Gibco), 200 ng/ml epidermal growth factor (catalog number RKP01133, Reprokine, Tampa, FL, USA), and 2 ng/ml heparin (catalog number H3149-10KU, Sigma-Aldrich, Oakville, ON) on Matrigel-coated (catalog number 354230, Corning, Guelph, ON) tissue culture plates at 37 °C with 5% CO_2_ as previously described^51^. ReN VM cells were differentiated into co-cultures of neurons and astrocytes in DMEM/F12, supplemented with 2% N21-MAX, 2 ng/ml glial-derived neurotrophic factor (catalog number PHC7045, Gibco), and 500 μM dibutyryl-cyclic-adenosine monophosphate (catalog number D0627-250MG, Sigma-Aldrich) as previously described^29^. ReN *MAPT*^−/−^ cells were generated using a paired Cas9 nickase design based on two gRNAs with the sequences ‘atcacttcgaactcctggcg’ and ‘cacgctgggacgtacgggtt’. The gRNAs were designed using CRISPR design tool (crispr.mit.edu) and selected based on high specificity scores and proximity to the ATG start codon site. ReN cells were plated in 6-well plates at 337,500 cells/well 24 h before being transfected with EditPro. The ratio of plasmids used was 6 Cas9 nickase: 1 gRNA: 1 gRNA for a total of 2 ug DNA and 4 ul of EditPro transfection reagent. Cells were expanded and diluted for clonal selection.

Two clones, validated by genetic sequencing and western blotting, were isolated.For inhibition of ATPase function in non-muscle myosin heavy chains, cells were treated with 10 μM of active (S)-Blebbistatin (catalog number 13013-1, Cedarlane, Burlington, ON) or the inactive (R)-Blebbistatin enantiomer (catalog number 13165-1, Cedarlane) for 2 h.

The study did not involve human subjects. All animal work was undertaken in accordance with an animal use protocol (number 4183.4) approved by the animal care committee at the University Health Network, Toronto, ON. Transgenic Tau P301L mice with strain name Thy1-hTau.P301L and non-transgenic mice used were both of FVB/N genetic background and sacrificed at 2 years of age. Mouse brain lysates were collected in 0.5% NP-40, 0.25% deoxycholate, 150 mM Tris-HCl (pH 7.5), 5 mM EDTA, 10 mM NaF, 1 mM Na orthovanadate, 1 mM PMSF, 1x cOmplete protease inhibitor cocktail (catalog number 11836170001, Roche Canada, Mississauga, ON) and 1x PhosSTOP phosphatase inhibitor cocktail (catalog number 4906837001, Roche). The lysates were then homogenized with three 1-minute long pulses of bead-beating with 1 min of cooling at 4 °C between each pulse. Supernatants were collected after centrifugation at 20,000 g for 15 min at 4 °C.

### Plasmid preparations

All site-directed mutagenesis and polymerase chain reactions were performed with the Q5 Site-Directed Mutagenesis and Hot Start High-Fidelity 2X Master Mix kits, respectively (catalog numbers E0554S and M0494S, New England Biolabs, Ipswich, MA). All ligation reactions were performed with the T4 DNA ligase (catalog number M0202S, New England Biolabs) after vector backbones had been dephosphorylated with Fast AP thermosensitive alkaline phosphatase (catalog number EF0651, Thermo Fisher Scientific). All plasmids were transformed into 5-alpha Competent *E*. *coli* (catalog number C2987H, New England Biolabs). All plasmids were purified using the PureLink HiPure Plasmid Filter Maxiprep Kit (catalog number K2100-16, Thermo Fisher Scientific). The foundation lox construct and the inducible construct were derived from a pre-existing ‘Puro-Cas9’ donor vector for targeting a tetracycline-inducible Cas9 cassette to the human *AAVS1*/PPP1R12C locus (catalog number 58409, Addgene, Cambridge, MA, RRID:Addgene_58409). The Flpo construct was derived from the pDIRE plasmid (catalog number 26745, Addgene, RRID:Addgene_26745) and the paavCAG-iCre construct was purchased directly (catalog number 51904, Addgene, RRID:Addgene_51904). The donor vectors were originally gifts from Danwei Huangfu^52^, Rolf Zeller^53^, and Jinhyn Kim^54^, respectively.

#### gRNAs

The gRNAs were cloned into the MLM3636 vector (a gift from Keith Joung, catalog number 43860, Addgene, RRID:Addgene_43860) using BsmBI (catalog number R0580S, New England Biolabs) as previously described^55^. Oligonucleotide pairs used to generate gRNAs are listed (Fig. S1).

#### Foundation cassette (FC)

Site-directed mutagenesis was used to insert lox71, lox2272, and frt sites, mutate gRNA PAM sequences in the homology arms, and insert unique restriction enzyme sites BbvCI and AsiSI. BbvCI (catalog number SCC008, New England Biolabs) and AsiSI (catalog number R0630S, New England Biolabs) were subsequently used to insert the Kan^R^ coding sequence in place of the Puro^R^ coding sequence from the original vector. The Kan^R^ coding sequence was derived from the Tau1-441-EGFP plasmid (a gift from Dr. George S. Bloom)^56^ after it was first amplified by PCR using primers with the respective restriction sites.

#### Inducible expression cassette (IEC)

The CAG promoter and the rtTA_3_ coding sequence were PCR amplified from paavCAG-iCre and pTRIPZ (a gift from Drs. Christopher Boehm and Peter St George-Hyslop, University of Toronto, ON, Canada), respectively. They were ligated together using BamHI (catalog number FD0054, Thermo Fisher Scientific) and reamplified by PCR to attach restriction sites BspTI (catalog number FD0834, Thermo Fisher Scientific) and Bsu15I (catalog number FD0144, Thermo Fisher Scientific) at the 5’ and 3’ ends, respectively. The coding sequence of Tau-EGFP was amplified from the Tau1-441-EGFP plasmid with restriction sites BbvCI and AsiSI attached at the 5’ and 3’ ends, respectively, and it was cloned into the ‘Puro-Cas9’ donor backbone downstream of the TREtight sequence in place of the original Cas9. The vector, now containing Tau-EGFP, was PCR amplified to attach restriction sites BspTI and Bsu15I before it was ligated to CAG-rtTA_3_. Site-directed mutagenesis was used to insert lox66, lox2272, and frt sites, delete Tau to make the EGFP-only control construct, delete the second repeat domain in Tau to make 3 R Tau-EGFP, and to mutate CCG to CTG at residue 301 in Tau to create the Tau^P301L^-EGFP mutant.

### Transfection

IMR-32 cells were trypsinized and plated at 75% confluency in 6-well plates 1 day before transfection using the jetPRIME reagent (catalog number 114-07, Polyplus, Illkirch, France) according to the manufacturer’s protocol. For inserting the foundation cassette (FC) using CRISPR-Cas9 nickase gene editing, cells were transfected at a ratio of 6 Cas9n: 1 gRNA: 1 gRNA: 5 FC expressing plasmids to a total mass of 3.5 μg of plasmids per well. Cells were then selected with 1 mg/mL G418 (catalog number 11811031, Thermo Fisher Scientific) and clonally expanded. For the swap-in of the inducible expression cassette, cells were transfected at a ratio of 1 iCre: 4 IEC plasmids to a total mass of 3.0 μg/well. Cells were then selected in separate wells at a range of puromycin (P7255-25MG, Sigma-Aldrich) concentrations spanning 600 ng/ml to 1.2 μg/ml. IMR-32 cells were transfected at a ratio of 1: 3. ReN VM cells were detached with Accutase (catalog number A1110501, Gibco) and plated at 67,500 cells/well in Matrigel-coated 24-well plates 1 day before transfection. Cells were then transfected at 250 ng DNA/well using the same ratios as above with 1 μL/well of EditPro stem transfection reagent (catalog number GST-2174, MTI-GlobalStem). For the selection of gene-edited cells, 1 mg/ml G418 (for FC positives) or 0.3 μg/ml puromycin (for IEC positives) were added to the cell culture medium and surviving cells were clonally expanded.

### Genomic PCR

Genomic DNA was extracted from clones grown on 12-well plates using the PureLink Genomic Mini Kit (catalog number K182001, Invitrogen). Precise transgene insertion into the target site at the *AAVS1* locus was determined by genomic PCR using 25 ng of genomic DNA amplified for 28 cycles using ‘ggaactctgccctctaacgc’ and ‘acccaatatcaggagactaggaagg’ as the forward and reverse primers, respectively. PCR products were purified with a gel/PCR extraction kit (catalog number DF300, Froggabio, ON, Canada). Genomic PCR of ReN *MAPT*^−/−^ cells was performed with 25 ng of genomic DNA using the primer pair ‘atggagcacgggatgagga’ and ‘ccccttggcttgcagtgat’ at an annealing temperature of 66 °C for 28 cycles.

### Immunoprecipitation

For immunoprecipitation of EGFP fusion proteins, inducible IMR-32 clone 27-3 cells were treated with 2.0 µg/mL doxycycline (catalog number DB0889-25G, BioBasic, ON, Canada) for durations indicated in the main text and figures before cell lysis. Inducible ReN VM cells were treated with 3.0 µg/mL doxycycline and 0.3 µg/mL puromycin for their respective durations before lysis. The lysis buffer consisted of 0.5% NP-40, 0.25% deoxycholate, 150 mM Tris-HCl (pH 7.5), 5 mM EDTA, 10 mM NaF, 1 mM Na orthovanadate, 1 mM PMSF, 1x cOmplete protease inhibitor cocktail (catalog number 11836170001, Roche Canada, Mississauga, ON) and 1x PhosSTOP phosphatase inhibitor cocktail (catalog number 4906837001, Roche). Lysate from each biological replicate was centrifuged at 3,000 g for 5 min at 4 °C before incubation with 20 µL (if used for mass spectrometry analysis downstream, otherwise 7 µL per biological replicate) of agarose-conjugated GFP nanotrap slurry (catalog number gta-10, Chromotek, Planegg-Martinsried, Germany) on a turning wheel at 4 °C for 2 h. Beads were then washed twice in buffer consisting of 450 mM NaCl and 150 mM Tris-HCl (pH7.5), once in buffer consisting of 150 mM NaCl and 150 mM Tris-HCl (pH 7.5), once in 25 mM HEPES (pH 7.5), and once in 10 mM HEPES (pH 7.5) before acid elution. Bound proteins were eluted consecutively with 100 μL and 200 μL of elution buffer comprised of 20% acetonitrile and 0.2% trifluoroacetic acid (pH 1.9).

For immunoprecipitation of DJ-1, cells were induced for durations indicated, collected in the aforementioned lysis buffer, centrifuged at the same settings, and incubated with 5 µL of DJ-1 antibody/biological replicate overnight on a turning wheel at 4 °C. Lysates were then incubated with 5 µL of Protein A sepharose beads per biological replicate (catalog number 101041, Invitrogen) for 4 h to capture the antibodies. After 3 washes in buffer consisting of 150 mM NaCl and 150 mM Tris-HCl (pH 7.5), captured proteins were eluted with 1x BOLT LDS sample buffer (catalog number B0007, Invitrogen) at 70 °C.

### Western blot

Cells were lysed in the same buffer used for immunoprecipitation. After protein concentrations were determined by BCA (catalog number 23227, Thermo Fisher Scientific) and made equal, 35 to 75 µg total protein per lane were loaded onto 4–12% Bis-Tris BOLT SDS-PAGE gels (catalog number NW04125BOX, Invitrogen) in MOPS running buffer (catalog number B0001, Invitrogen). Proteins were then transferred onto a 0.45 µm PVDF membrane (catalog number IPVH00010, Millipore) at 45 V for 1 h 15 min in Tris-glycine transfer buffer containing 20% methanol. Proteins were incubated with the appropriate antibody overnight at 4 °C. The membrane was then rinsed in TBST before incubation with HRP-linked anti-mouse or anti-rabbit IgG antibodies. Chemiluminescence was detected with Amersham ECL Prime Western Blotting Detection Reagent (catalog number RPN2232, GE Healthcare Canada, Mississauga, ON). Band intensities on Western blots were quantified with ImageJ (RRID: SCR_003070) and then analyzed and graphed with GraphPad Prism 7.

### Live cell microscopy

Cells were cultured on 4-well Nunc Lab-Tek II chambered cover-glass plates (Thermo Fisher Scientific) at 67,500 cells/well and induced for 12 h with 2.0 μg/ml of doxycycline before being imaged on a Leica DMI 6000 B fluorescence microscope (Leica Microsystems Canada, Richmond Hill, ON).

### Immunocytochemistry

ReN cells were grown on coverslips (catalog number 83.1840.002, Sarstedt) and differentiated using the PreD protocol for one week. Cells were rinsed twice with PBS, fixed in 4% formaldehyde (catalog number NC0268594, Electron Microscopy Sciences, Hatfield, PA, USA) for 10 min at 37 °C, rinsed again twice with PBS, and then permeabilized in 0.25% Triton X-100 (catalog number TRX506.500, Bioshop) for 5 min at room temperature. Next, cells were blocked in 3% goat serum (catalog number 16210064, Gibco) for 30 min at room temperature before being incubated with anti-TuJ1 (used at 1:500 dilution) and anti-GFAP (used at 1:500 dilution) overnight at 4 °C. The next day, cells were rinsed three times in PBS, incubated with Alexa Fluor 488 goat anti-mouse IgG (H + L) (Catalog number A11001, Gibco; used at 1:1000 dilution) and Alexa Fluor 568 goat anti-rabbit IgG (H + L) (catalog number A11011, Gibco; used at 1:1000 dilution) for 1 h at room temperature. Cells were then rinsed three times in PBS, mounted in ProLong® Diamond Antifade Mountant with DAPI (catalog number P36965, Gibco) and imaged with 200x final magnification on a Leica DMI 6000 B fluorescence microscope using the DAPI, FITC, and TRITC filters.

### Peptide preparation for mass spectrometry analyses

Immunoprecipitation eluates were suspended in 500 mM triethylammonium bicarbonate 9 M urea, made to 8 mM tris carboxyethyl phosphine, then heated to 60 °C for 30 min. Each sample was treated for 1 hour in 16 mM 4-vinylpyridine. Trypsin digestion (2 micrograms per sample) was allowed to proceed for 16 hours at 37 °C, then peptides were isobarically labelled with 8-plex iTRAQ reagents (catalog number 4390812, Sciex, Concord, ON) according to the manufacturer’s protocol. Mixed sets of iTRAQ-labelled trypsin-digested immunoprecipitates were diluted in aqueous 0.1% (v/v) trifluoroacetic acid, then bound to C18 Bond Elut micro columns (catalog number A57003100, Agilent Technologies, Santa Clara, CA, USA), before being washed with aqueous 0.1% (v/v) trifluoroacetic acid and released in 50% acetonitrile. The acetonitrile was removed on a centrifugal evaporator at 36 °C and the peptides suspended in aqueous 0.1% (v/v) formic acid. The Titansphere Phos-TiO kit (catalog number 5010–21309, GL Sciences, Tokyo, Japan) was used according to the manufacturer’s instructions to purify phosphopeptides from the labelled sample mixtures. Phosphopeptide preparations were washed on C18 micro columns as described above.

### Quantitative mass spectrometry

The washed sample mixtures were delivered to an analytical column at 500 nL/min on an Easy-nLC 1000 HPLC system (Thermo Fisher Scientific) by the built-in autosampler, then separated at 300 nL/min with a binary mobile phase mixture of increasing acetonitrile concentration at a constant formic acid concentration of 0.1% (v/v). Over the course of each HPLC run, the acetonitrile content of the eluent was increased with linear gradients from 0 to 30% over 180 minutes, then from 30 to 100% over 60 minutes. The PepMap RSLC C18 stationary phase was 2 μm in diameter with 100 Å pores and was housed in a 25 cm long 75 μm diameter column (Thermo Fisher Scientific). The eluate was ionized at 2200 V and an ion source temperature of 275 °C by the nano-electrospray ionization source of an Orbitrap Fusion Tribrid instrument (Thermo Fisher Scientific).

The data acquisition cycle repeated continuously over each HPLC run and had an upper time limit of three seconds. It acquired a survey scan at a resolution setting of 60,000 in the orbitrap mass analyser of the instrument, followed by collision-induced dissociation of the most abundant multiply charged ions and their analysis in the linear ion trap mass analyser. The ten most abundant product ions were then synchronously further fragmented by high-energy collision induced dissociation at a relative energy of 65% and injected into the orbitrap mass analyser for mass analysis. An additional method using Orbitrap MS^2^ scans at a resolution setting of 30,000 following electron transfer dissociation was applied to phosphopeptide preparations. The m/z ranges in MS and MS^3^ scans were 400–1500 and 100–500 Th, respectively, while the MS^2^ scan range was variable under the normal scan rate setting. Isolation windows for MS^2^ and MS^3^ were 2 Th wide. Each m/z value for which three ion trap MS^2^ scans or four Orbitrap MS^2^ scans were acquired within a 20 second period was excluded from MS^2^ analysis for 100 minutes. The minimum precursor intensity required to trigger ion trap and Orbitrap MS^2^ were 50,000 and 100,000 counts respectively.

Protein sequence information was derived from the LC-MS data using the Mascot version 2.4.1 (Matrix Science, London, UK) and Sequest HT (Thermo Fisher Scientific) search engines within the Proteome Discoverer software version 1.4.0.288 (Thermo Fisher Scientific). The results from both algorithms were combined into a single data set, with quantifications and phosphosite estimation relying on the Reporter Ions Quantifier and PhosphoRS algorithms (version 3.0), respectively. An orthogonal search of the same LC-MS data was performed on PEAKS Studio 8 (Bioinformatics Solutions Incorporated, Waterloo, ON, Canada) using the PEAKS and De Novo search engines. For all three searches, 20 ppm precursor ion mass tolerance, 0.4 Da product ion mass tolerance, tryptic digestion with up to two missed cleavages and iTRAQ 8-plex labelling at lysine and amino terminal residues were fixed parameters. Glutamine and asparagine deamidation, cysteine pyridylethylation, methionine oxidation, cysteine dioxidation, cysteine trioxidation, as well as serine and tyrosine and threonine phosphorylation were specified as variable modifications in all searches. The mass shift equal to a diglycine modification of lysine side chains was the variable modification used to detect ubiquitylation. The Uniprot FASTA sequence database used contained entries for all human canonical and isoform protein variants (updated on December 20, 2015 and downloaded on March 1, 2016) as well as porcine trypsin, bovine serum albumin, *Lysobacter enzymogenes* Lysyl endopeptidase, 40 and 42 amino acid versions of human amyloid beta, wild-type Tau-EGFP, P301L Tau-EGFP and EGFP.

### Statistical analysis

Confidence levels were assigned to peptide-to-spectrum matches based on false discovery rate (FDR) estimation. FDR analysis was performed on the Percolator algorithm in Proteome Discoverer and by the decoy fusion method in PEAKS. Hierarchical clustering and ANOVA were performed on the PEAKS Quantitation node. The significance of the differential abundance measurements of candidate Tau interactors in wild-type versus P301L immunoprecipitates was evaluated by ANOVA at a *p-*value of 0.025. Only proteins identified and quantified by three or more unique peptides were subjected to ANOVA testing. For comparing enrichment of MYH10 in eluate samples, one-way ANOVA and Tukey’s post-hoc test was performed on three biological replicates per condition. For comparing levels of pS19 in myosin regulatory light chains in mouse brain homogenates, unpaired t-test and Tukey’s post-hoc test were performed on results from three biological replicates per condition. The comparison of MYH10 levels in ReN VM cells that either expressed or were ablated (*MAPT*^−/−^) in their expression of endogenous Tau was based on one-way ANOVA and Tukey’s post hoc-test. Levels of MYH10 in wild-type C57BL/6 mice and Tau^-/-^ mice were compared using unpaired Student’s t-test.

## Supplementary information

Supplementary Information

## Data Availability

The ProteomeXchange consortium^57^ PRIDE partner repository hosts the LC-MS data and search results under the dataset identifier PXD010040. Efforts to deposit plasmids to the AddGene global plasmid repository (Watertown, MA, USA) have been initiated. Cell models generated in this study will be made available to interested readers.
