# Supplementary material for: Tau interactome analyses in CRISPR-Cas9 engineered neuronal cells reveal ATPase-dependent binding of wild-type but not P301L Tau to non-muscle myosins
Source: Sci Rep. 2019 Nov 7;9:16238. doi: 10.1038/s41598-019-52543-5 (PMC6838314; doi:10.1038/s41598-019-52543-5)
Supplement: Supplementary file 1 — Supplementary Information [file 41598_2019_52543_MOESM1_ESM.pdf]

August 12, 2019

Supplementary Information to:

Tau interactome analyses in CRISPR-Cas9 engineered neuronal cells reveal ATPase-dependent binding of wild-type but not P301L Tau to non-muscle myosins

Xinzhu Wang[1,2], Declan Williams[1,2], Iris Müller[1], Mackenzie Lemieux[1], Ramona Dukart[2], Isabella BL Maia[1], Hansen Wang[1], Amanda L Woerman[3], Gerold Schmitt-Ulms[1,2]*

[1]Tanz Centre for Research in Neurodegenerative Diseases, University of Toronto, Ontario M5T 2S8, Canada.
[2]Department of Laboratory Medicine & Pathobiology, University of Toronto, Ontario M5S 1A8, Canada.
[3]Department of Neurology, University of California San Francisco, California 94158, USA.

*Corresponding author and lead contact: g.schmittulms@utoronto.ca, Krembil Discovery Tower, Room 6KD447, 60 Leonard Avenue, Toronto, Ontario M5T 0S8, Canada.

Supplementary Figure S1. gRNAs used for CRISPR-Cas9-mediated insertion of foundation cassette into AAVS1 locus.

Three separate gRNAs were tested for this application. Positive clones were obtained with gRNAs S3 and 27 in IMR cells and with gRNAs as2 and S3 in ReN cells.

Supplementary Figure S2. Detailed record of PSM assignments to Tau-EGFP.

Graph depicting the primary structure of Tau-EGFP, together with peptides identified in this study through PSMs that were interpreted by the PEAKS (Version 8) algorithm. In addition to providing a more comprehensive overview of PSMs observed, including numerous repeat assignments, the graph identifies PTMs (phosphorylations and ubiquitinations), as well as chemical modifications introduced deliberately (iTRAQ labeling and alkylation with 4-vinylpyridine) or inadvertently (e.g., methionine oxidation) during sample processing. Blue and grey lines demark peptide sequences that comprise at least one tryptic boundary or were mapped to Tau-EGFP based on the *de novo* sequencing feature incorporated in PEAKS, respectively.

Supplementary Figure S3. Comparison of Tau interactomes in IMR and ReN cells.

Entries quantified on the basis of at least six iTRAQ signature ion profiles, sorted alphabetically.

Supplementary Figure S4. Raw data of western blot panels shown in Fig. 1.

To facilitate comparisons of raw data with processed images subpanels are shown here with the same alphabetical labels as those shown in the main body of the manuscript. Note that in this and subsequent western blot panels samples derived from wild-type human Tau-EGFP expressing cells, P301L mutant Tau-EGFP expressing cells, and EGFP expressing cells are labeled as ‘W in black font’, ‘P’ in blue font and ‘G’ in green font, respectively.

Supplementary Figure S5. Raw data of western blot panels shown in Fig. 5. Subpanel labelling as in **Fig. 5** of main manuscript.

Supplementary Figure S6. Raw data of western blot panels shown in Fig. 7. Subpanel labelling as in **Fig. 7** of main manuscript.

Supplementary Figure S7. Raw data of western blot panels shown in Fig. 8. Subpanel labelling as in **Fig. 8** of main manuscript.

Figure S1

gRNAs used for *AAVS1* insertion of homology-directed repair template to generate foundation cassette-positive clones.

Identifier	Oligos with embedded gRNAs and restriction enzyme overhang*
as2	5’ACACCAGAACCAGAGCCACATTAACG 3’<br>3’GTCTTGGTCTCGGTGTAATTGCAAAA 5’
27	5’ACACCgACCCCACAGTGGGGCCACTAG 3’<br>3’GcTGGGGTGTCACCCCGGTGATCAAAA 5’
S3	5’ACACCGGGGCCACTAGGGACAGGATG 3’<br>3’GCCCCGGTGATCCCTGTCCTACAAAA 5’

* Invariant overhangs compatible with BsmBI restriction enzyme target sites are in blue, and gRNA sequences are underlined.

Figure S2: Tau sequence coverage and posttranslational modifications observed in IMR32 cells expressing 3R/4R wild-type Tau-EGFP

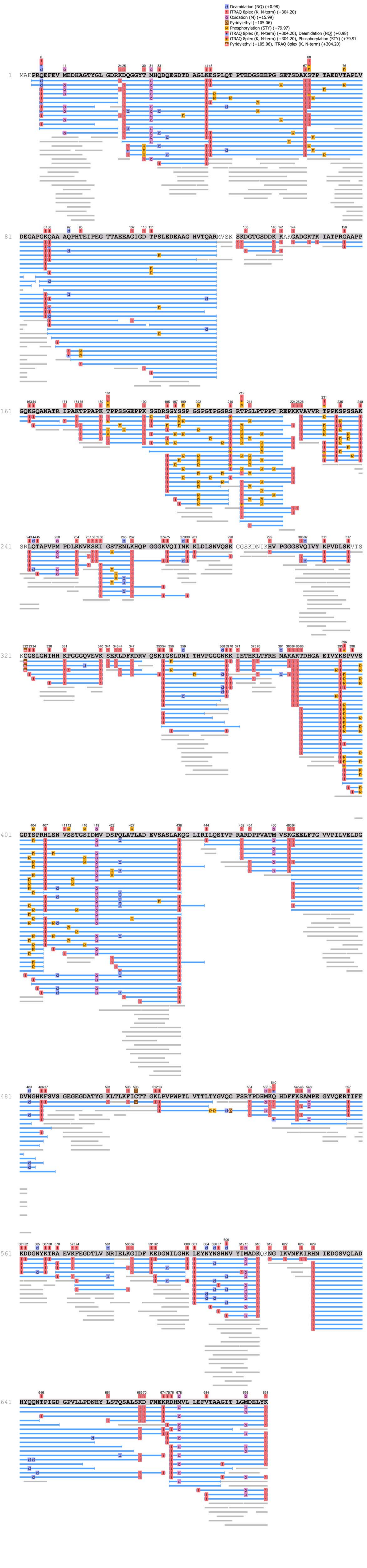

Figure S3

Accession	Description	Description (curated)	Gene Symbol	Coverage	# of Prot.	Unique Pept.	# of Pept.	# of PSMs	WT(1)/ P301L(3)	P301L(1)/ P301L(3)	WT(2)/ P301L(3)	P301L(2)/ P301L(3)	WT(3)/ P301L(3)	EGFP/ P301(3)	Count	Mascot Score	Sequest Score	WT(1)/ P301L(3)	P301L(1)/ P301L(3)	WT(2)/ P301L(3)	P301L(2)/ P301L(3)	WT(3)/ P301L(3)	EGFP/ P301(3)	Count	Mascot Score	Sequest Score	Rel. counts	Amino acids	MW [kDa]	pI
Q15029-2	Isoform 2 of 116 kDa U5 small nuclear ribonucleoprotein component OS=Homo sapiens GN=EFTUD2 - [U5S1_HUMAN]	116 kDa U5 small nuclear ribonucleoprotein component	EFTUD2	4.3%	3	2	3	6	0.571	0.747	0.671	0.717	1.124	0.151	6	14.0											-6	937	105.3	5.22
P31946-2	Isoform Short of 14-3-3 protein beta/alpha OS=Homo sapiens GN=YWHAB - [1433B_HUMAN]	14-3-3 protein beta/alpha	YWHAB	21.3%	8	1	5	48	0.833	0.610	0.832	0.652	1.069	0.019	21	87.4	20.3										-21	244	27.8	4.83
P62258	14-3-3 protein epsilon OS=Homo sapiens GN=YWHAE PE=1 SV=1 - [1433E_HUMAN]	14-3-3 protein epsilon	YWHAE	46.3%	6	8	11	156	0.840	0.573	0.808	0.694	1.101	0.047	65	319.5	362.4										-65	255	29.2	4.74
P61981	14-3-3 protein gamma OS=Homo sapiens GN=YWHAG PE=1 SV=2 - [1433G_HUMAN]	14-3-3 protein gamma	YWHAG	26.7%	7	3	7	52	0.843	0.610	0.832	0.652	1.069	0.019	21	100.3	25.7										-21	247	28.3	4.89
P27348	14-3-3 protein theta OS=Homo sapiens GN=YWHAQ PE=1 SV=1 - [1433T_HUMAN]	14-3-3 protein theta	YWHAQ	58.4%	8	8	12	152	0.835	0.593	0.843	0.747	1.119	0.068	69	389.7	601.2										-69	245	27.7	4.78
P63104	14-3-3 protein zeta/delta OS=Homo sapiens GN=YWHAZ PE=1 SV=1 - [1433Z_HUMAN]	14-3-3 protein zeta/delta	YWHAZ	43.7%	11	6	9	102	0.874	0.644	0.884	0.710	1.102	0.187	41	252.2	457.4										-41	245	27.7	4.79
Q13442	28 kDa heat- and acid-stable phosphoprotein OS=Homo sapiens GN=PDAP1 PE=1 SV=1 - [HAP28_HUMAN]	28 kDa heat- and acid-stable phosphoprotein	PDAP1	22.1%	1	3	3	26	1.007	0.721	1.246	0.811	2.347	0.255	12	50.4	151.8										-12	181	20.6	8.87
P52815	39S ribosomal protein L12, mitochondrial OS=Homo sapiens GN=MRPL12 PE=1 SV=2 - [RM12_HUMAN]	39S ribosomal protein L12, mitochondrial	MRPL12	35.4%	2	5	5	20	1.156	0.804	1.050	0.683	1.427	0.047	14	74.7											-14	198	21.3	8.87
Q99714	3-hydroxyacyl-CoA dehydrogenase type-2 OS=Homo sapiens GN=HSD17B10 PE=1 SV=3 - [HCD2_HUMAN]	3-hydroxyacyl-CoA dehydrogenase type-2	HSD17B10	49.0%	2	6	6	39	0.542	0.671	0.675	0.801	0.835	0.124	17	99.5	207.9										-17	261	26.9	7.78
P46783	40S ribosomal protein S10 OS=Homo sapiens GN=RPS10 PE=1 SV=1 - [RS10_HUMAN]	40S ribosomal protein S10	RPS10	55.2%	5	11	11	248	0.905	0.587	0.959	0.731	1.259	0.049	117	470.1	886.2										-117	165	18.9	10.15
P62280	40S ribosomal protein S11 OS=Homo sapiens GN=RPS11 PE=1 SV=3 - [RS11_HUMAN]	40S ribosomal protein S11	RPS11	44.3%	2	8	8	149	0.567	0.574	0.642	0.752	0.860	0.024	51	274.6	59.2										-51	158	18.4	10.3
P25398	40S ribosomal protein S12 OS=Homo sapiens GN=RPS12 PE=1 SV=3 - [RS12_HUMAN]	40S ribosomal protein S12	RPS12	31.8%	1	4	4	80	0.730	0.536	0.617	0.741	1.061	0.043	26	181.8	69.5										-26	132	14.5	7.21
P62277	40S ribosomal protein S13 OS=Homo sapiens GN=RPS13 PE=1 SV=2 - [RS13_HUMAN]	40S ribosomal protein S13	RPS13	53.6%	3	10	10	157	0.885	0.647	0.846	0.722	1.086	0.035	66	318.1	481.5										-66	151	17.2	10.54
P62263	40S ribosomal protein S14 OS=Homo sapiens GN=RPS14 PE=1 SV=3 - [RS14_HUMAN]	40S ribosomal protein S14	RPS14	37.8%	3	6	6	84	0.766	0.565	0.795	0.711	1.030	0.043	42	245.3	839.7										-42	151	16.3	10.05
P62841	40S ribosomal protein S15 OS=Homo sapiens GN=RPS15 PE=1 SV=2 - [RS15_HUMAN]	40S ribosomal protein S15	RPS15	60.0%	8	5	5	138	0.996	0.566	1.057	0.817	1.240	0.103	90	562.2											-90	145	17	10.39
P62244	40S ribosomal protein S15a OS=Homo sapiens GN=RPS15A PE=1 SV=2 - [RS15A_HUMAN]	40S ribosomal protein S15a	RPS15A	49.2%	2	6	6	137	0.667	0.667	0.641	0.664	0.961	0.050	44	295.7	570.2										-44	130	14.8	10.13
M0R210	40S ribosomal protein S16 OS=Homo sapiens GN=RPS16 PE=1 SV=1 - [M0R210_HUMAN]	40S ribosomal protein S16	RPS16	38.8%	5	7	7	128	0.623	0.637	0.591	0.786	0.867	0.048	66	225.7	140.1										-66	129	14.4	10.23
P08708	40S ribosomal protein S17 OS=Homo sapiens GN=RPS17 PE=1 SV=2 - [RS17_HUMAN]	40S ribosomal protein S17	RPS17	66.7%	4	11	11	222	0.758	0.582	0.879	0.789	1.015	0.064	103	583.0	675.4										-103	135	15.5	9.85
P62269	40S ribosomal protein S18 OS=Homo sapiens GN=RPS18 PE=1 SV=3 - [RS18_HUMAN]	40S ribosomal protein S18	RPS18	55.9%	2	12	12	225	0.757	0.591	0.797	0.680	1.145	0.051	98	420.2	710.2										-98	152	17.7	10.99
[illegible]	[illegible]	[illegible]	[illegible]	[illegible]	[illegible]	[illegible]	[illegible]	[illegible]	[illegible]	[illegible]	[illegible]	[illegible]	[illegible]	[illegible]	[illegible]	[illegible]	[illegible]	[illegible]	[illegible]	[illegible]	[illegible]	[illegible]	[illegible]	[illegible]	[illegible]	[illegible]	[illegible]	[illegible]	[illegible]	[illegible]

Figure S4

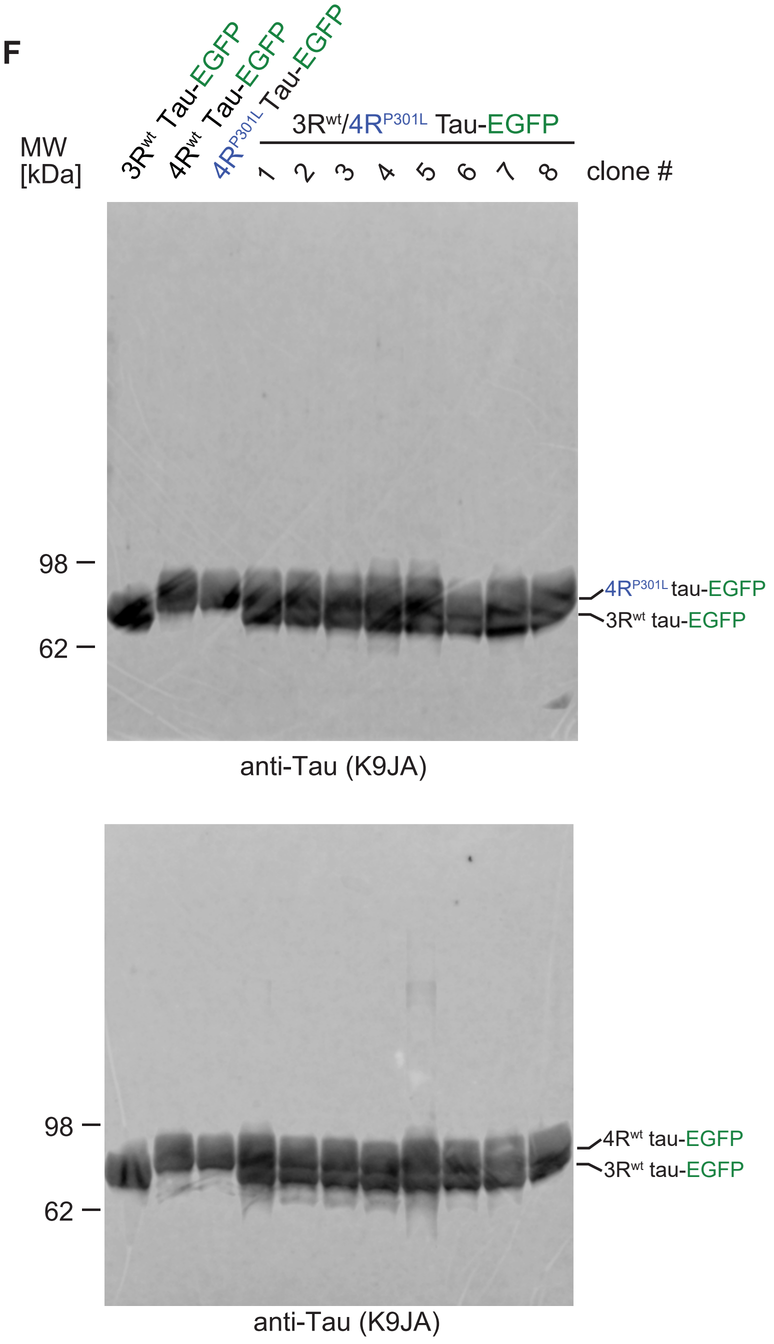

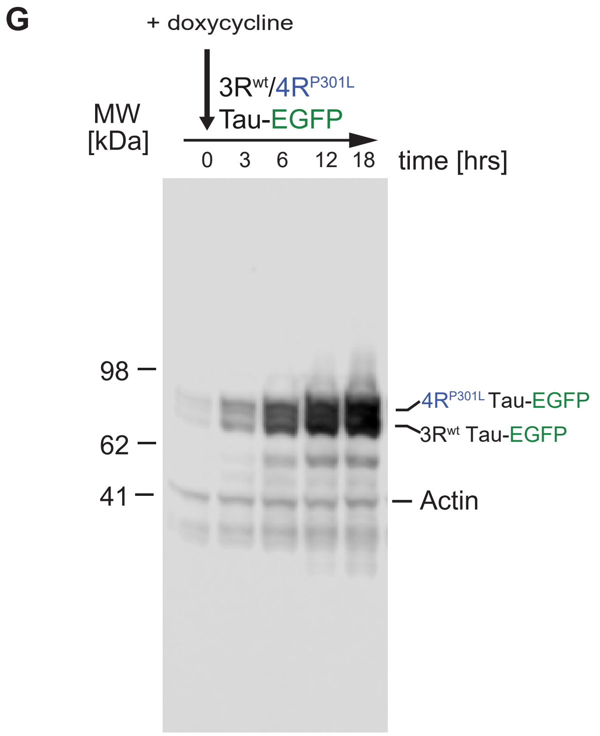

Note: Tau and Actin sginals were probed on the same western blot.

Figure S5

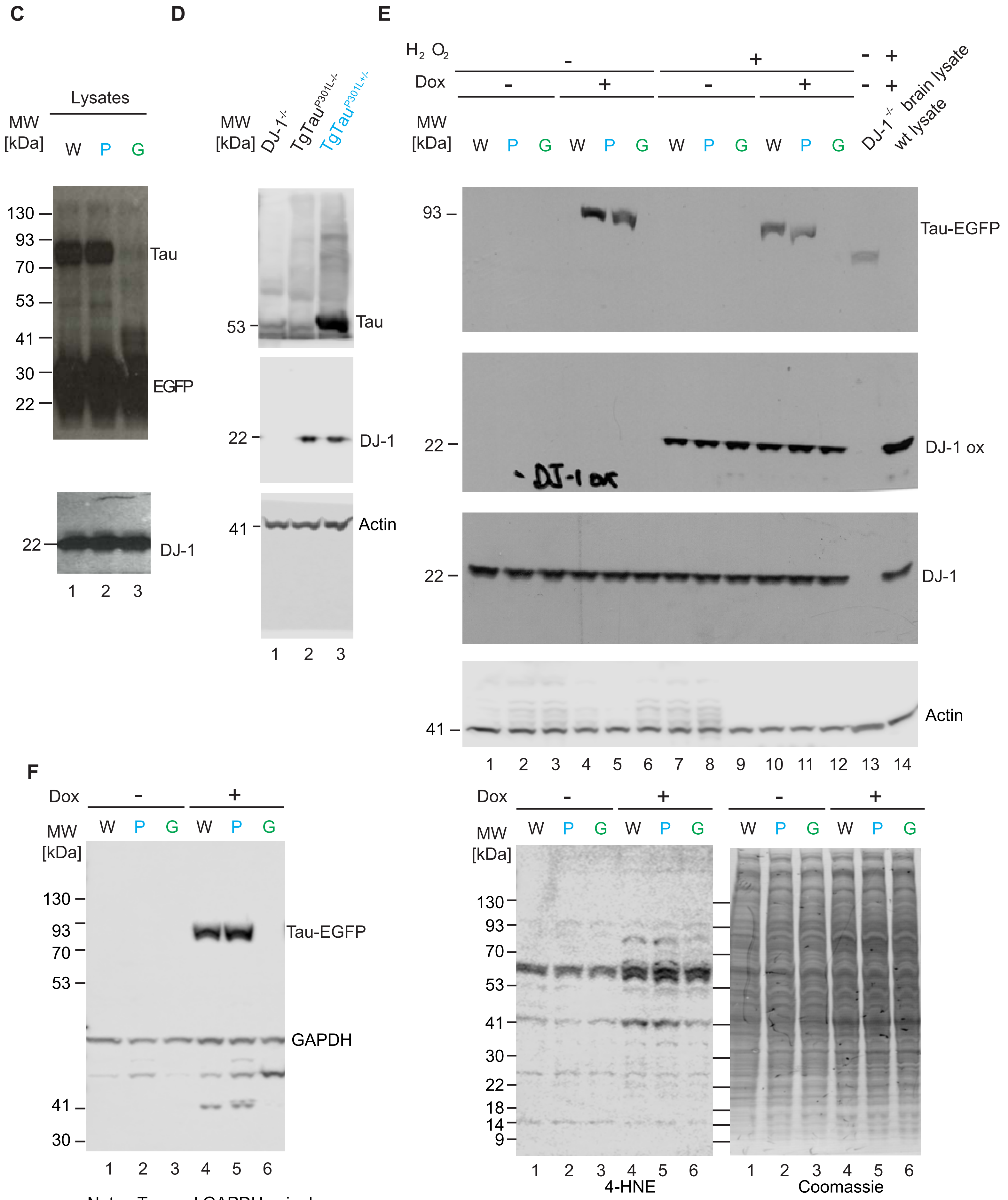

Note: Tau and GAPDH sginals were probed on the same western blot.

Figure S6

E

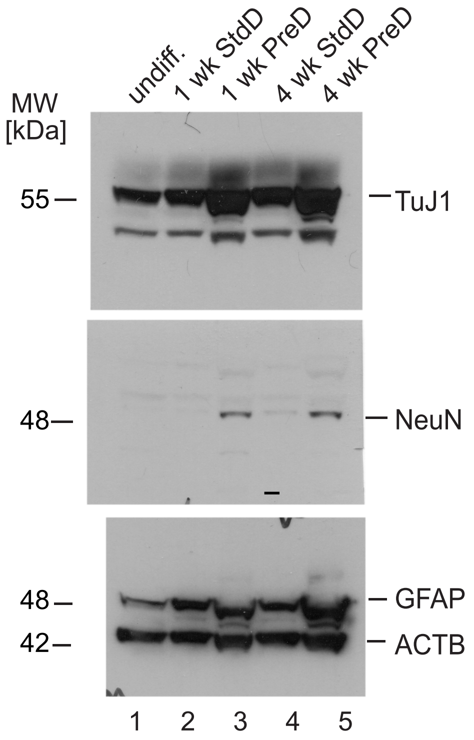
undiff.
1 wk StdD
1 wk PreD
4 wk StdD
4 wk PreD
MW [kDa]
55
TuJ1
48
NeuN
48
42
GFAP
ACTB
1 2 3 4 5

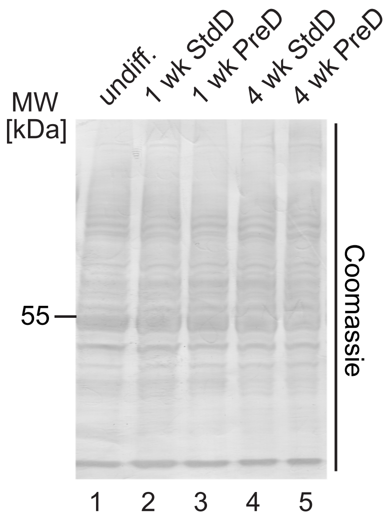
undiff.
1 wk StdD
1 wk PreD
4 wk StdD
4 wk PreD
MW [kDa]
55
Coomassie
1 2 3 4 5

F

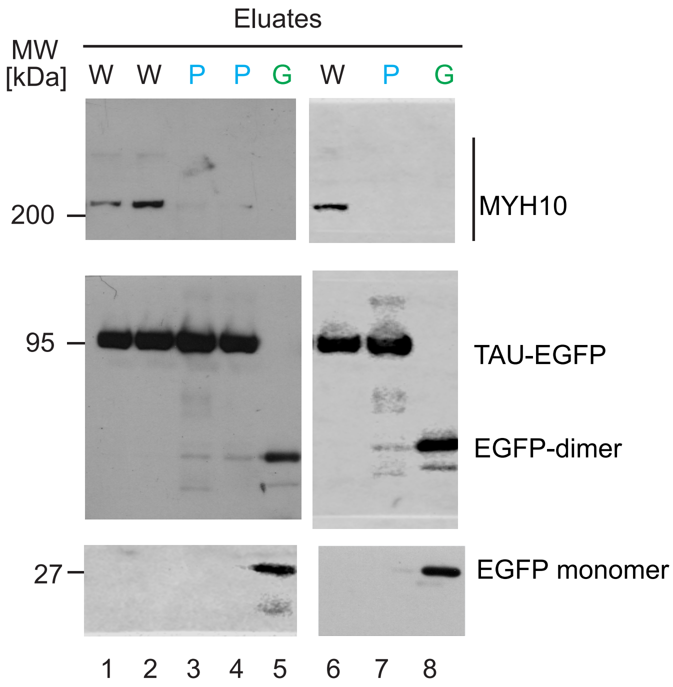
Eluates
MW [kDa]
W W P P G W P G
200
MYH10
95
TAU-EGFP
EGFP-dimer
27
EGFP monomer
1 2 3 4 5 6 7 8

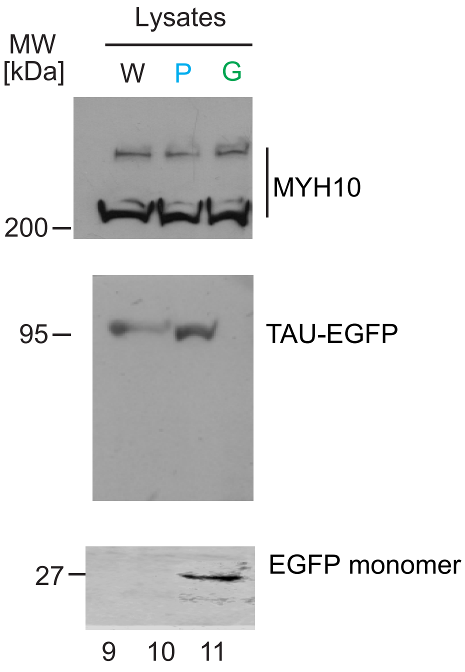
Lysates
MW [kDa]
W P G
MYH10
200
95
TAU-EGFP
27
EGFP monomer
9 10 11

Figure S7

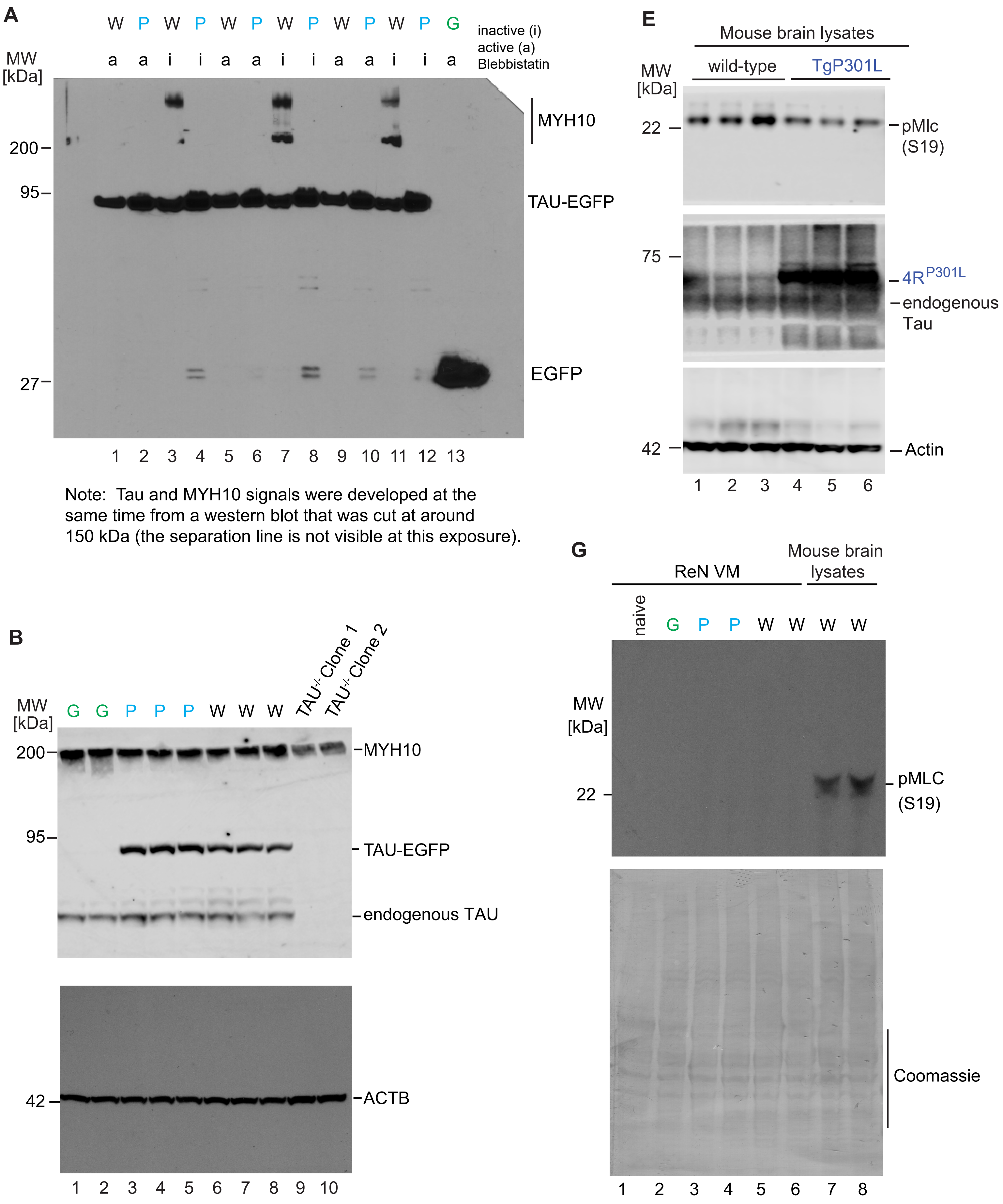
A
W P W P W P W P W P W P G
a a i i a a i i a a i i a
inactive (i)
active (a)
Blebbistatin
MW [kDa]
200
95
27
MYH10
TAU-EGFP
EGFP
1 2 3 4 5 6 7 8 9 10 11 12 13
Note: Tau and MYH10 signals were developed at the same time from a western blot that was cut at around 150 kDa (the separation line is not visible at this exposure).
B
MW [kDa]
G G P P P W W W
TAU-/- Clone 1
TAU-/- Clone 2
200
95
42
MYH10
TAU-EGFP
endogenous TAU
ACTB
1 2 3 4 5 6 7 8 9 10
E
Mouse brain lysates
wild-type
TgP301L
MW [kDa]
22
75
42
pMlc (S19)
4R P301L
endogenous Tau
Actin
1 2 3 4 5 6
G
ReN VM
Mouse brain lysates
naive
G P P W W W W
MW [kDa]
22
pMLC (S19)
Coomassie
1 2 3 4 5 6 7 8
